# DNA-Compatible Suzuki-Miyaura Cross-Coupling Reaction of Aryl Iodides With (Hetero)Aryl Boronic Acids for DNA-Encoded Libraries

**DOI:** 10.3389/fchem.2022.894603

**Published:** 2022-06-14

**Authors:** Vijay Kumar Siripuram, Yashoda Krishna Sunkari, Thu-Lan Nguyen, Marc Flajolet

**Affiliations:** Laboratory of Molecular and Cellular Neuroscience, The Rockefeller University, New York, NY, United States

**Keywords:** suzuki-miyaura cross-coupling reaction, palladium catalysis, C-C bond formation, DNA-encoded library (DEL), drug discovery

## Abstract

An efficient method for the C-C bond formation via water soluble Na_2_PdCl_4_/sSPhos mediated Suzuki-Miyaura cross-coupling reaction of DNA-conjugated aryl iodide with (het)aryl boronic acids has been developed. This reaction proceeds at 37°C in water and acetonitrile (4:1) system. We also demonstrated that numerous aromatic and heteroaromatic boronic acids of different electronic natures, and harboring various functional groups, were highly compatible providing the desired coupling products in good to excellent yields. This DNA-compatible Suzuki-Miyaura cross-coupling reaction has strong potential to construct DNA-Encoded Libraries (DELs) in the context of drug discovery.

## Introduction

DNA-Encoded Library (DEL) technology is based on the concept from Brenner and Lerner (Brenner and Lerner 1992) and it is commonly used in the pharmaceutical industry to identify novel chemical matter that binds and modulates specific protein targets (Melkko et al., 2004; Melkko et al., 2007; Clark et al., 2009; Kleiner et al., 2011; Franzini et al., 2014; Salamon et al., 2016; Goodnow et al., 2017; Favalli et al., 2018; Neri and Lerner 2018; Ottl et al., 2019; Yuen et al., 2019; Zhao et al., 2019; Kunig et al., 2021; Shi et al., 2021). During the past decade, the use of DEL technology provided a great opportunity to identify drug-like compounds that can bind selectively to a variety of target proteins (Deng et al., 2012; Gentile et al., 2012; Disch et al., 2013; Samain et al., 2015; Seigal et al., 2015; Harris et al., 2016; Belyanskaya et al., 2017; Dawadi et al., 2020; Chamakuri et al., 2021). More recently, a number of different powerful applications leveraging the DEL technology have been proposed (Huang et al., 2022; Sunkari et al., 2022; Zhao et al., 2022). To expand the chemical space of these DNA-Encoded Libraries, a greater variety of DNA-Compatible reactions is required. Although much progress has been made in this direction (Potowski et al., 2021; Nie et al., 2022; Shen et al., 2022; Yang et al., 2022), the availability of efficient synthetic methods for the synthesis of DELs remains an important challenge.

A DEL is a complex mixture composed of a large number of drug-like molecules in which each molecule is conjugated to a unique and specific DNA-oligomer that encodes its chemical structure. Due to the presence of DNA barcodes, and due to the process of generating a DEL that involves alternation of chemical and molecular steps (e.g., split-and-pool strategy), any chemical modification has to be performed in the presence of DNA. This implicates that chemical reaction conditions must be mild and compatible with aqueous conditions. Although progress has been made in this direction, the availability of efficient methods for the synthesis of DELs remains an important challenge.

**SCHEME 1:**
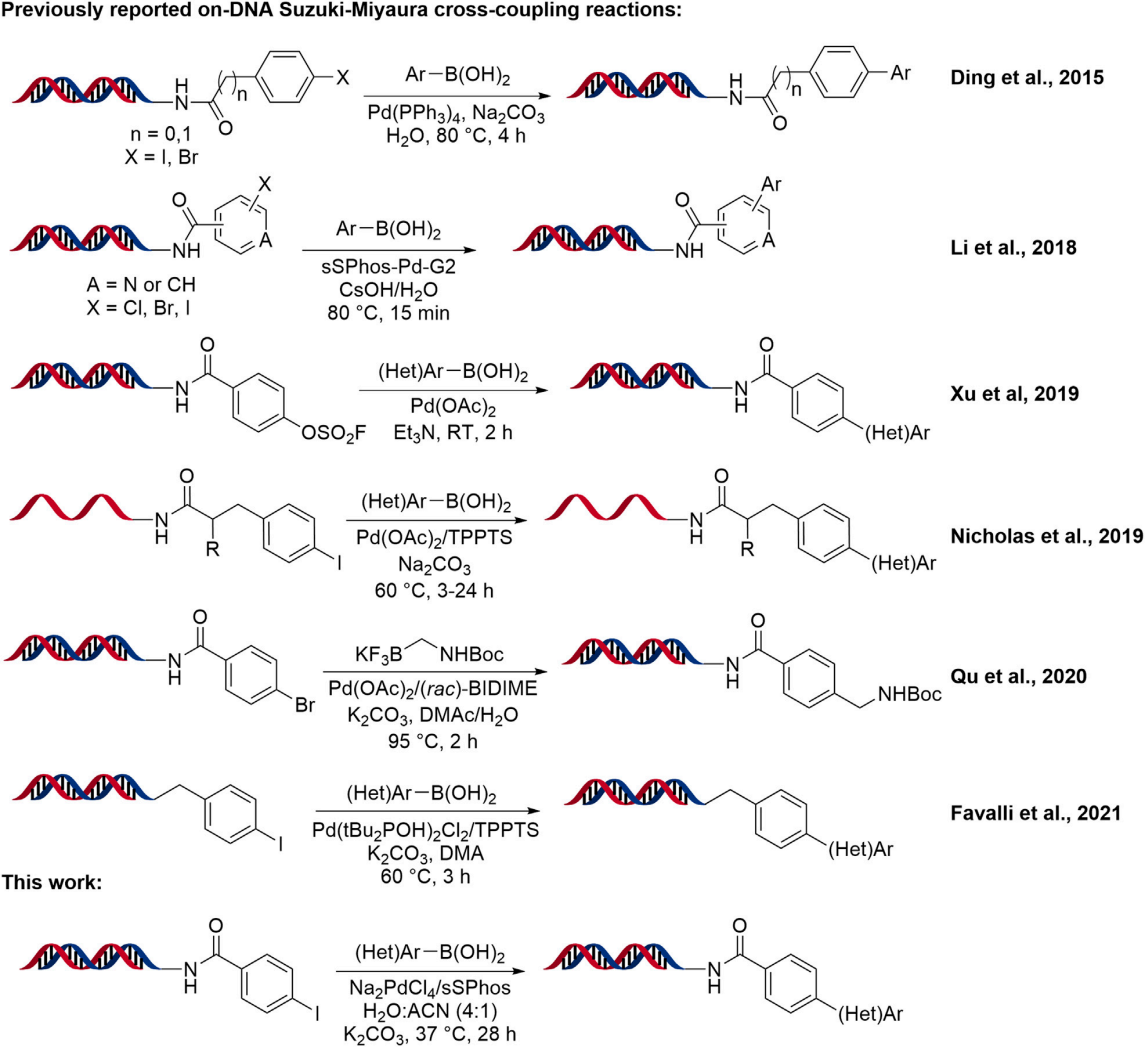
On-DNA Suzuki-Miyaura Cross-Coupling Reaction Development.

During the last few years, the interest in DNA-compatible transition metal catalyzed cross-coupling reactions has increased, and especially for C-C bond formation using Pd-catalyzed Suzuki-Miyaura cross-coupling reaction (Miyaura and Suzuki 1995; Bellina et al., 2004; Martin and Buchwald 2008). Due to mild reaction conditions, commercial availability of coupling partners and high chemo-selectivity, the Suzuki–Miyaura cross-coupling reaction is now the second most utilized reaction in the field of medicinal chemistry (Brown and Bostrom 2016), after the amide bond formation reaction. While methods for the Suzuki-Miyaura cross-coupling reaction were reported in the context of DNA-Encoded Library synthesis (Scheme 1) (Omumi et al., 2011; Deng et al., 2015; Ding and Clark 2015; Litovchick et al., 2015; Ding et al., 2016a; Ding et al., 2016b; Li and Huang 2018; Nicholas et al., 2019; Xu et al., 2019; Qu et al., 2020; Favalli et al., 2021), there is a great need to further develop this reaction.

In 2011, Omumi et al. reported first Suzuki-Miyaura cross-coupling reaction in the presence of DNA (C8-Ar-G-modified oligonucleotides) using aryl boronic acids employing Pd(OAc)_2_ and a hydrophilic phosphine ligand, 3-tri (3-sulfonatophenyl)phosphine trisodium (TPPTS) (Omumi et al., 2011). In 2014, Ding et al. reported first Suzuki-Miyaura cross-coupling reaction using Pd(PPh_3_)_4_ under aqueous conditions in the context of DEL (Ding and Clark 2015). Later, the same group reported the reaction for less reactive DNA-conjugated aryl chlorides using a combination of phosphinous acid/Pd catalyst and the sSPhos ligand at 80°C (Ding et al., 2016a). Compared with Pd(PPh_3_)_4_, this catalyst system is better for the coupling of pyrimidinyl chloride and unreactive aryl chloride with challenging heteroaryl boronates. In 2015, two reports published the construction of 334 and 34.7 million-membered DELs synthesized in three cycles, in which they introduced boronic acid/ester building blocks by Suzuki cross-coupling in the second and the third cycle respectively (Deng et al., 2015; Litovchick et al., 2015). In 2016, Ding et al. reported the construction of 3.5 million-membered DEL in three cycles, in which they introduced Suzuki-Miyaura cross-coupling reaction in the second cycle (Ding et al., 2016b). Li et al. developed a robust Suzuki-Miyaura reaction protocol employing a water-soluble Pd-precatalyst for the coupling of DNA-linked aryl halides with a wide range of boronic acids/esters including heteroaryl boronates (Li and Huang 2018). Additionally, Nicholas et al. reported an alternative Pd(OAc)_2_ catalyzed DNA-compatible reaction with aromatic, heteroaromatic, and vinyl boronic acids at 60°C (Nicholas et al., 2019). Recently, Xu et al. also reported Suzuki-Miyaura cross-coupling reaction on-DNA with aryl fluorosulfonates as electrophiles at room temperature (Xu et al., 2019). Very recently Qu et al. developed a Pd-mediated Suzuki-Miyaura cross-coupling of DNA-conjugated aryl bromides with potassium Boc-protected aminomethyltrifluoroborate at 95°C (Qu et al., 2020). Most recently Favalli et al. reported the Suzuki-Miyaura cross-coupling reaction of DNA-conjugated aryl iodides with (het)aryl boronic acids at 70°C using Pd(OAc)_2_ and TPPTS (Favalli et al., 2021).

Despite these few reports that are mostly using high temperature conditions, there is a need to develop this reaction at relatively low temperature to avoid DNA degradation. It is well established that DNA is highly stable in physiological conditions (37°C or at lower temperature). Importantly, among those studies, only one study reported Suzuki-Miyaura cross-coupling reaction on-DNA, at room temperature, using a non-readily available coupling partner (aryl fluorosulfonate) (Xu et al., 2019). Here, we introduced a DNA friendly method performed at room temperature using water soluble Na_2_PdCl_4_/ sSPhos mediated Suzuki-Miyaura cross-coupling reaction. For that purpose, DNA-conjugated aryl iodide was used with over fifty boronic acids that are readily available and full DNA integrity was confirmed by mass spectrometry.

## Results and Discussion

The Suzuki-Miyaura cross-coupling of DNA-conjugated aryl iodide **1a** with phenyl boronic acid **2a** in 4:1 water and acetonitrile system was first examined to optimize reaction conditions for synthesizing the DNA-conjugated biaryl product **3a**. Previously, Pd(OAc)_2_/N-XantPhos catalysts that led to excellent yield in the cross-coupling of (hetero)aryl halides with boronic acids was examined (Wang et al., 2015). However, it did not result in the formation of **3a** at 37°C after 24 h (Table 1, entry 1). Previously reported reaction conditions (Favalli et al., 2021, Li and Huang, 2018, Ding and Clark, 2015) were tested for this coupling reaction at 37°C (Table 1, entry 2-4), Pd(OAc)_2_ and sSPhos-Pd-G2 did not furnish the desired product, whereas Pd(PPh_3_)_4_ gave 41% yield at 37°C. Interestingly, when water soluble Pd catalyst Na_2_PdCl_4_ (20 equiv), N-XantPhos ligand (40 equiv) and K_2_CO_3_ were used, the product yield was 61% (Table 1, entry 5). On the other hand, when N-XantPhos was replaced with sSPhos, the desired product could be isolated with a 67% yield under the same conditions. (Table 1, entry 6). These results clearly indicate that the water soluble Pd catalyst might have a dramatic influence on this coupling reaction. This speculation was corroborated by the observation that Na_2_PdCl_4_ gave the good yield, while Pd(OAc)_2_and sSPhos-Pd-G2 gave no product formation. Noteworthy, when sSPhos was replaced with X-Phos and XantPhos, a decrease in yield was observed (Table 1, entries 7–8).

**TABLE 1:** Optimization of Suzuki-Miyaura Cross-Coupling Reaction*^a^*.

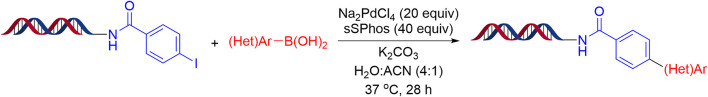				
S.No	Pd catalyst (20 eq)	Ligand (40 eq)	Base	Solvent	Yield%
1	Pd(OAc)_2_	N-XantPhos	K_2_CO_3_ (300 eq)	DMF:H_2_O (4:1)	0%
2	Pd(OAc)_2_	TPPTS	K_2_CO_3_ (500 eq)	H_2_O:DMA (1:1)	0%
3	sSPhos-Pd-G2	–	CsOH (400 eq)	H_2_O:DMA:Dioxane	0%
4	Pd(Ph_3_)_4_	–	Na_2_CO_3_ (40 eq)	H_2_O:DMA:ACN	41%
5	Na_2_PdCl_4_	N-XantPhos	K_2_CO_3_ (300 eq)	H_2_O:ACN (4:1)	61%
6	Na_2_PdCl_4_	sSPhos	K_2_CO_3_ (300 eq)	H_2_O:ACN (4:1)	67%
7	Na_2_PdCl_4_	X-Phos	K_2_CO_3_ (300 eq)	H_2_O:ACN (4:1)	54%
8	Na_2_PdCl_4_	XantPhos	K_2_CO_3_ (300 eq)	H_2_O:ACN (4:1)	55%
9	Na_2_PdCl_4_	sSPhos	Na_2_CO_3_ (300 eq)	H_2_O:ACN (4:1)	50%
10	Na_2_PdCl_4_	sSPhos	Cs_2_CO_3_ (300 eq)	H_2_O:ACN (4:1)	45%
11	Na_2_PdCl_4_	sSPhos	K_3_PO_4_ (300 eq)	H_2_O:ACN (4:1)	41%
12	Na_2_PdCl_4_	sSPhos	CsOH (300 eq)	H_2_O:ACN (4:1)	48%
13	Na_2_PdCl_4_	sSPhos	KOH (300 eq)	H_2_O:ACN (4:1)	62%
14	Na_2_PdCl_4_	sSPhos	K_2_CO_3_ (300 eq)	H_2_O:ACN (4:1)	81%^b^
15	Na_2_PdCl_4_	sSPhos	K_2_CO_3_ (600 eq)	H_2_O:ACN (4:1)	94%^c^
16	Na_2_PdCl_4_	sSPhos	K_2_CO_3_ (1500 eq)	H_2_O:ACN (4:1)	80%
17	Na_2_PdCl_4_	sSPhos	K_2_CO_3_ (300 eq)	H_2_O:ACN (4:1)	69%^d^
18	Na_2_PdCl_4_	sSPhos	K_2_CO_3_ (600 eq)	H_2_O:ACN (1:1)	91%
19	Na_2_PdCl_4_	sSPhos	K_2_CO_3_ (600 eq)	H_2_O:DMSO (4:1)	73%^c^
21	Na_2_PdCl_4_	sSPhos	K_2_CO_3_ (600 eq)	H_2_O:DMF (4:1)	72%^c^
21	Na_2_PdCl_4_	sSPhos	K_2_CO_3_ (600 eq)	H_2_O:DMA (4:1)	76%^c^
22	Na_2_PdCl_4_	sSPhos	K_2_CO_3_ (600 eq)	H_2_O:Dioxane (4:1)	89%^c^
23	Na_2_PdCl_4_	sSPhos	K_2_CO_3_ (600 eq)	H_2_O:THF (4:1)	75%^c^

a Reaction Conditions: 1 equiv of **1a** (1 mM in Borate Buffer pH 9.5, 250 mM), 200 equiv of boronic acid (200 mM in ACN/H_2_O, 1:1), 20 equiv Na_2_PdCl_4_, 40 equiv sSPhos (10 mM in H_2_O), 300 equiv K_2_CO_3_, H_2_O:ACN (4:1), 37^o^C for 24 h.

b Reaction time 28 h.

c 600 equiv K_2_CO_3_.

d 10 equiv Na_2_PdCl_4_, 20 equiv sSPhos (5 mM in H_2_O).

After identifying Na_2_PdCl_4_/sSPhos as the best catalyst, we examined different bases. This base screening revealed that Na_2_CO_3_, Cs_2_CO_3_, K_3_PO_4_, and CsOH gave poor yields, while KOH gave a comparable yield to K_2_CO_3_ (Table 1, entries 9–13). Next, using K_2_CO_3_ and by increasing the reaction time to 28 h, the yield increased to 81% (Table 1, entry 14). Doubling the amount of base (600 equiv) also increased the product yield and reached 94% (Table 1, entry 15). However, a further increase of the amount of base (1,500 equiv) led to a decrease in the product yield. Attempt to reduce the catalyst loading did not reach complete conversion anymore (Table 1, entries 16-17). When the percentage of solvent mixture was altered to be 1:1 (water and acetonitrile), a slight decrease of the yield was observed (Table 1, entry 18). Further solvent screening revealed that DMSO, DMF, DMA and THF gave lower yields, and 1,4-dioxane solvent gave a comparable yield (Table 1, entries 19–23).

Therefore, we concluded that the optimal condition is: 20 equiv of Pd catalyst, 40 equiv of ligand as the catalyst system and K_2_CO_3_ (600 equiv) as the base, 4:1 water and acetonitrile, at 37°C for 28 h.

**SCHEME 2:**
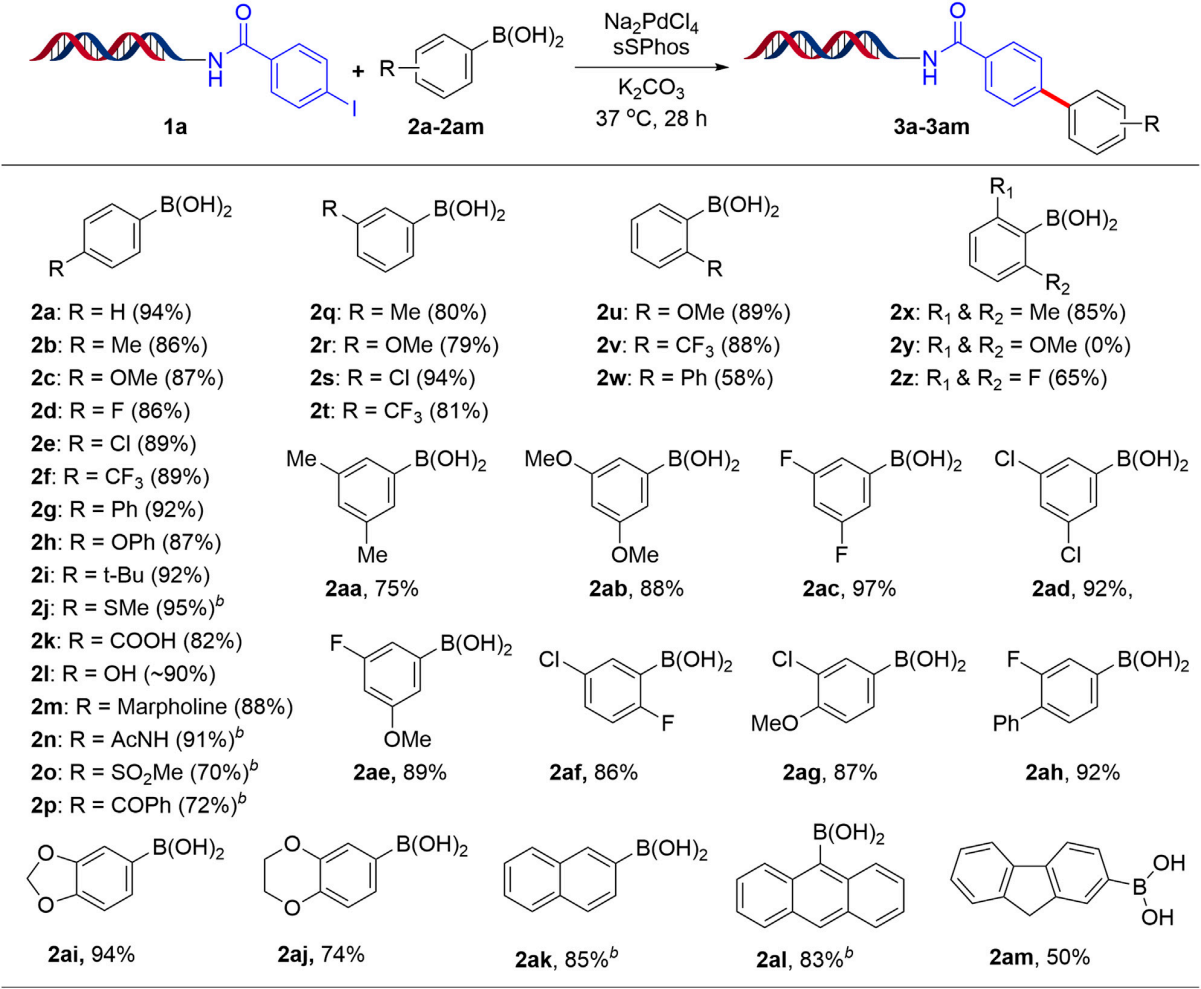
Suzuki-Miyaura cross-coupling reaction of aryl boronic acids with 1a^*a*^. ^a^Reaction Conditions: 1 equiv of 1a (1 mM in H_2_O), 200 equiv of aryl boronic acid (200 mM in ACN/H_2_O, 1:1), 20 equiv Na_2_PdCl_4_, 40 equiv sSPhos (10 mM in DMA), K_2_CO_3_, H_2_O:ACN (4:1), 37^o^C for 28 h; ^b^H_2_O:1,4-dioxane (4:1).

We next explored the substrate scope using the present optimized protocol and the results are summarized in Scheme 2. As expected, a number of aryl boronic acids bearing electron-rich, electron-deficient groups and functional groups at the para-position worked well, providing coupling products **3a-3p** with 86–94% yields. Remarkably, some ortho- and meta-substituted aryl boronic acids were also applicable, leading to the formation of **3q-3w** products with good yields. Sterically hindered 2,6-disubstituted phenyl boronic acids **3x** and **3z** afforded good to excellent conversion except **3y** possibly due to bulky methoxy groups. Furthermore, 3,4-disubstituted, 3,5-disubstituted, 2,4-disubstituted and 2,5-disubstituted aryl boronic acids with electron-rich, electron-deficient groups also gave excellent yields (**3aa-3aj**). Additionally, coupling reaction of 2-naphthyl, 9-anthracenyl, fluorene-2-boronic acids proceeded smoothly to deliver the coupling products **3ak-3am** respectively.

**SCHEME 3:**
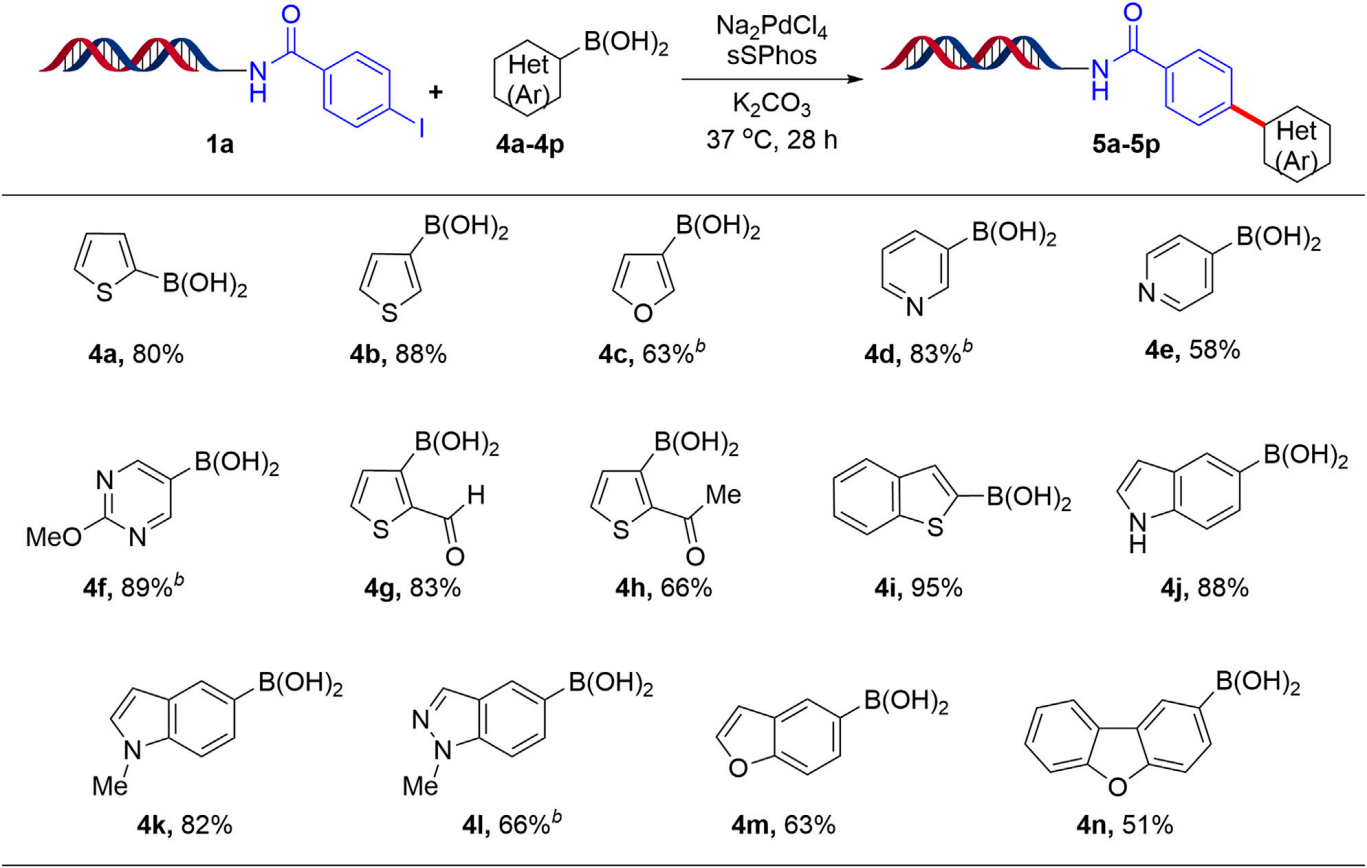
Suzuki-Miyaura cross-coupling reaction of heteroaryl boronic acids with 1a^*a*^. ^a^Reaction Conditions: 1 equiv of 1a (1 mM in H_2_O), 200 equiv of heteroaryl boronic acid (200 mM in ACN/H_2_O, 1:1), 20 equiv Na_2_PdCl_4_, 40 equiv sSPhos (10 mM in DMA), K_2_CO_3_, H_2_O:ACN (4:1), 37°C for 28 h; ^b^H_2_O:1,4-dioxane.

Finally, after successful implementation of this protocol for the coupling of DNA-conjugated aryl iodide (**1a**) with aryl boronic acids, we next focused on the coupling of **1a** with heteroaryl boronic acids. The results are summarized in Scheme 3. Thus, thiophene, furan, pyridyl and pyrimidyl boronic acids yielded the respective products (**5a-5h**) with good to excellent conversion (63–89%). The versatility of this methodology was further demonstrated by coupling **1a** with fused heterocycles such as benzothiophene, indole, N-methyl indole, indazole, benzofuran and dibenzofuran boronic acids to give the respective coupling products (**5i-5n**) in moderate to excellent yields (51–95%). Furthermore, the coupling of DNA-conjugated aryl bromide with phenyl boronic acid is also compatible with the Suzuki-Miyaura reaction (41%). However, the coupling of DNA-conjugated aryl chloride with phenyl boronic acid yielded only 3%.

## Conclusion

In summary, we have developed an efficient method for the coupling of aryl iodide conjugated on double-stranded DNA with (het)aryl boronic acids via water soluble Na_2_PdCl_4_/sSPhos mediated Suzuki-Miyaura cross-coupling reaction. This reaction proceeds at 37°C in water and acetonitrile (4:1) system. These results demonstrate the scope of the Suzuki-Miyaura cross-coupling reaction for on-DNA substrates. The present protocol displays broad substrate scope and tolerates the functionality that would be very useful for construction of DNA-encoded libraries.

## Data Availability

The original contributions presented in the study are included in the article/Supplementary Material, further inquiries can be directed to the corresponding authors.
