# Supplementary material for: DNA-Compatible Suzuki-Miyaura Cross-Coupling Reaction of Aryl Iodides With (Hetero)Aryl Boronic Acids for DNA-Encoded Libraries
Source: Front Chem. 2022 Jun 14;10:894603. doi: 10.3389/fchem.2022.894603 (PMC9237475; doi:10.3389/fchem.2022.894603)
Supplement: Supplementary file 1 [file DataSheet1.docx]

***Supporting Information***

DNA Compatible Suzuki-Miyaura Cross-Coupling Reaction of Aryl Iodides with (Hetero)Aryl Boronic Acids for DNA-Encoded Libraries

**Vijay Kumar Siripuram^1^**^*^, **Yashoda Krishna Sunkari^1^, Thu-Lan Nguyen^1^ and Marc Flajolet^1^**^*^

^1^Laboratory of Molecular and Cellular Neuroscience, 1230 York Avenue, The Rockefeller University, New York, USA

* Corresponding Authors: Marc Flajolet ([marc.flajolet@rockefeller.edu](mailto:marc.flajolet@rockefeller.edu)) and Vijay Kumar Siripuram ([vijaykumar.siripuram@rockefeller.edu](mailto:vijaykumar.siripuram@rockefeller.edu))

**Material and Method**

**Reagents:**

Reagents commercially available: Acetonitrile (HPLC grade, cat# 34851, Sigma-Aldrich, USA), *N*,*N*-dimethyl formamide (DMF) (HPLC grade, cat# 588725, Sigma-Aldrich, USA), *N*,*N*-dimethyl acetamide (DMA) (HPLC grade, 99.5%, cat# 22916, Alfa Aesar, USA), 1,4-dioxane (HPLC grade, cat# 296309, Sigma-Aldrich, USA), Dimethyl sulfoxide (DMSO) (≥99.5%, cat# D5879, Sigma-Aldrich, USA), 1,1,1,3,3,3-Hexafluoroisopropyl alcohol (99.9%, cat# 00080, Chem-Impex international, Inc., USA), Triethylamine (≥99%, cat# T0886, Sigma-Aldrich, US), K_2_CO_3_ (≥99%, cat# 209619, Sigma-Aldrich, US). UltraPure distilled water (DNAse, RNAse free, cat# 10977-015, Invitrogen, USA) and Sodium hydroxide solution (BioUltra, 10 M in H_2_O, cat# 72068, Sigma-Aldrich, USA) were used for buffer preparation. Deionized water was used for LCMS mobile phase preparation.

**Design and preparation of DNA:**

The DNA used in the present work was designed in-house based on previous head pieces (MW=6,765), custom made and synthetized by Integrated DNA Technologies, Inc. (IDT, Coralville, Iowa, USA). Lyophilized DNA samples were resuspended in Tris-EDTA (TE) buffer pH 8.0 at 1 mM, tested for quality purpose by mass spectrometry (LC/MS) and stored at -20°C.

**LCMS Instrumentation, acquisition conditions and data analysis:**

LCMS analyses were performed using an Agilent LCMS system (LCMS-TOF 6230B) (Agilent, Santa Clara, CA, USA) according to the manufacturer instructions consisting of LC parts, a multisampler (model number - G7167A), binary pump (model number -G7112B), column compartment (model number -G7116A) and UV/MWD detector (model number - G7165A), and MS TOF (model number - G6230B).

**Analysis Conditions:**

The mobile phase consisted of 100 mM HFIP and 8.9 mM TEA in deionized water (A) and MeOH (B). The samples were injected onto a reverse phase chromatography column (Targa C18, 5 μm, 50 x 2.1 mm, 120 A^°^), and gradient elution was as follows: 1% B hold for 1 minute; 1%-95% B for 12 minutes and set the post time for 3 minutes to equilibrate; at a flow rate of 0.4 mL/min and the column temperature at 40 °C. The Dual ESI negative mode polarity was used with scan range of 500-3200 Da. The source conditions were as follows: Drying gas flow 12L/min at 325 °C and a nebulizer pressure of 30 psi. The capillary voltage was set to 4000V.

**Data acquisition and analysis:**

The data for each DNA sample were acquired using Agilent mass hunter workstation data acquisition software and the data were analyzed using Agilent mass hunter qualitative analysis B.07.00. The quality and estimated yield of DNA samples were determined by examination of the UV absorbance traces at 260 nm and Total Ion Chromatogram (TIC) traces corresponding to the peaks.

**Synthesis of DNA Conjugated Aryl iodides (1a):**

To the DNA (1 mM in 150 mM pH 9.5 borate buffer, 200 µL, 1 equiv) in a 1.5 mL safe-lock Eppendorf tube, 4-Iodobenzoic acid (200 mM, 100 µL, 100 equiv in DMSO) and DMT.MM (200 mM, 100 µL, 100 equiv in H_2_O) were added and the reaction mixture was stirred on ThermoMixer C at 900 RPM at 25 °C for ON. The reaction mixture was precipitated with EtOH and washed with ice cold 30% water and ethanol mixture and dried for 3 hrs. The pellet was resuspended in 200 μL H_2_O and treated with 10% piperidine for 2 h to remove the DMT.MM adducts. Again, the reaction mixture was precipitated with EtOH and washed with ice cold 30% water and ethanol mixture and dried. The pellet was resuspended in 200 μL pH 9.5 borate buffer and confirmed with the LCMS analysis (1 µL DNA + 6 µL H_2_O and 5 µL injected).

**Suzuki-Miyaura Coupling of DNA Conjugated Aryl Iodides with (Het)Aryl Boronic Acids:**

To **1a** (1 μL, 1 mM in borate buffer pH 9.5), was added 200 equiv. of (hetero)aromatic boronic acids (1 μL, 200 mM in water and acetonitrile) 20 equiv. of Na_2_PdCl_4_ (1 μL, 20 mM in water) and 40 equiv. of sSPhos (1uL, 40 mM in water and acetonitrile) followed by 600 equiv. of K_2_CO_3_ (2 μL, 300 mM in water). The mixture was vortexed and stood at 37 °C for 30 hours. After reaction, 30 equiv. of scavenger sodium diethyldithiocarbamic acid (compared with Na_2_PdCl_4_, 6 μL 1 M in ddH2O) were added to the mixture, and the reaction mixture was heated at 80 °C for 30 minutes. The mixture was centrifuged at 4 °C for 30 min at 12,000 rpm, and the resultant supernatant was collected. Add 5 M NaCl solution (10% by volume) and cold ethanol (2.5 times by volume, ethanol stored at -20 °C) to the resultant supernatant. The mixture was vortexed and incubated at -80 °C for at least 30 minutes. The sample was centrifuged for 30 minutes at 4°C in a microcentrifuge at 12,000 rpm to remove the supernatant. The resulting pellet was dissolved in ddH_2_O (30 μL) for LC-MS detection.


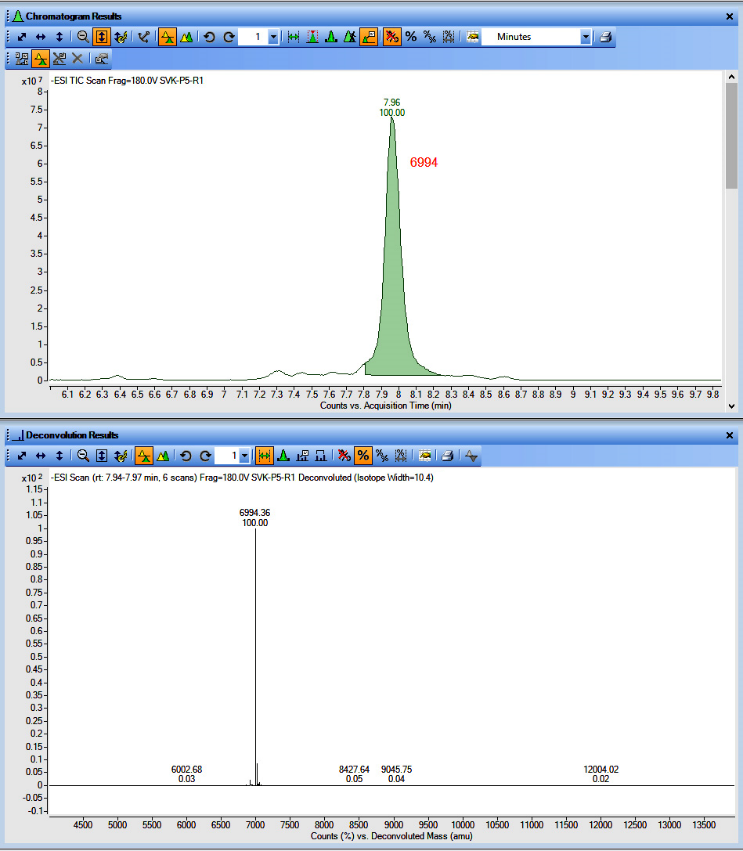

**Figure S1.** TIC and Deconvoluted mass spectrum of **1a**, expected: 6994; observed 6994.


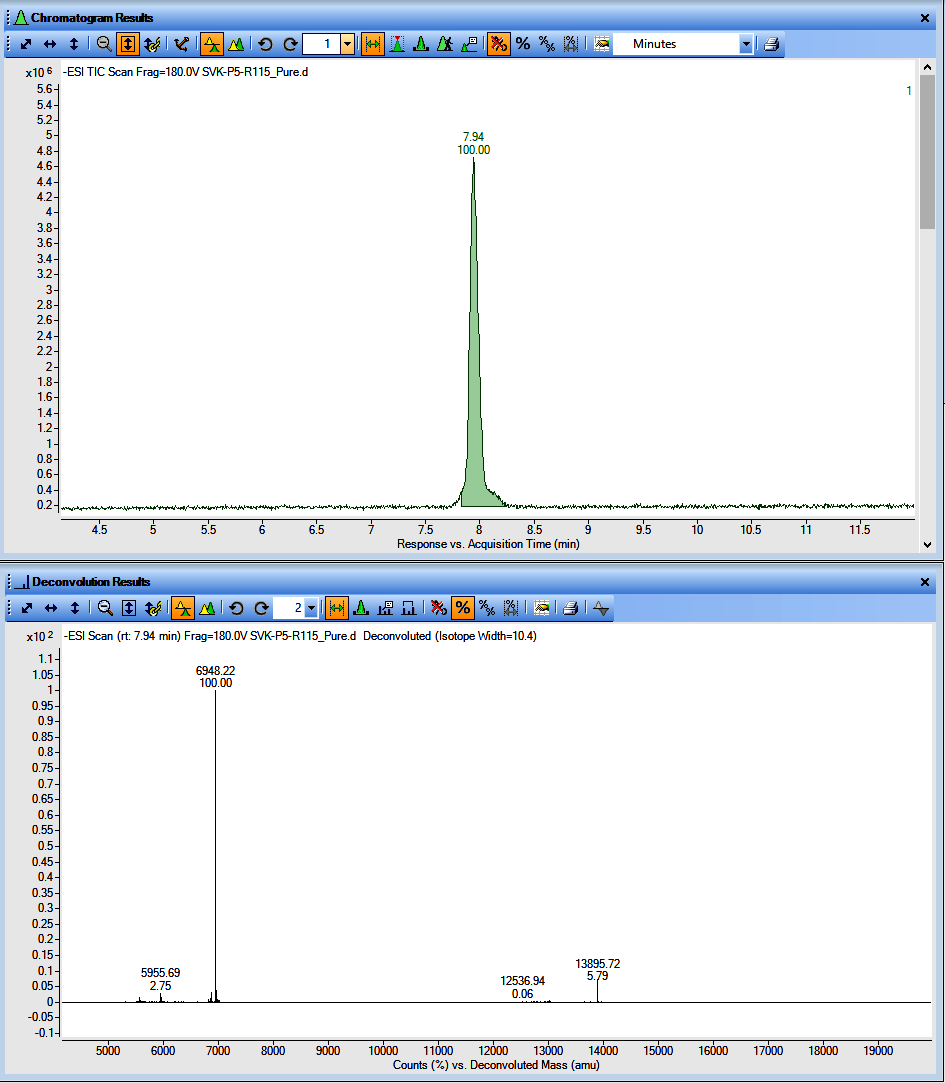

**Figure S2.** TIC and Deconvoluted mass spectrum of **1b**, expected: 6948; observed 6948.


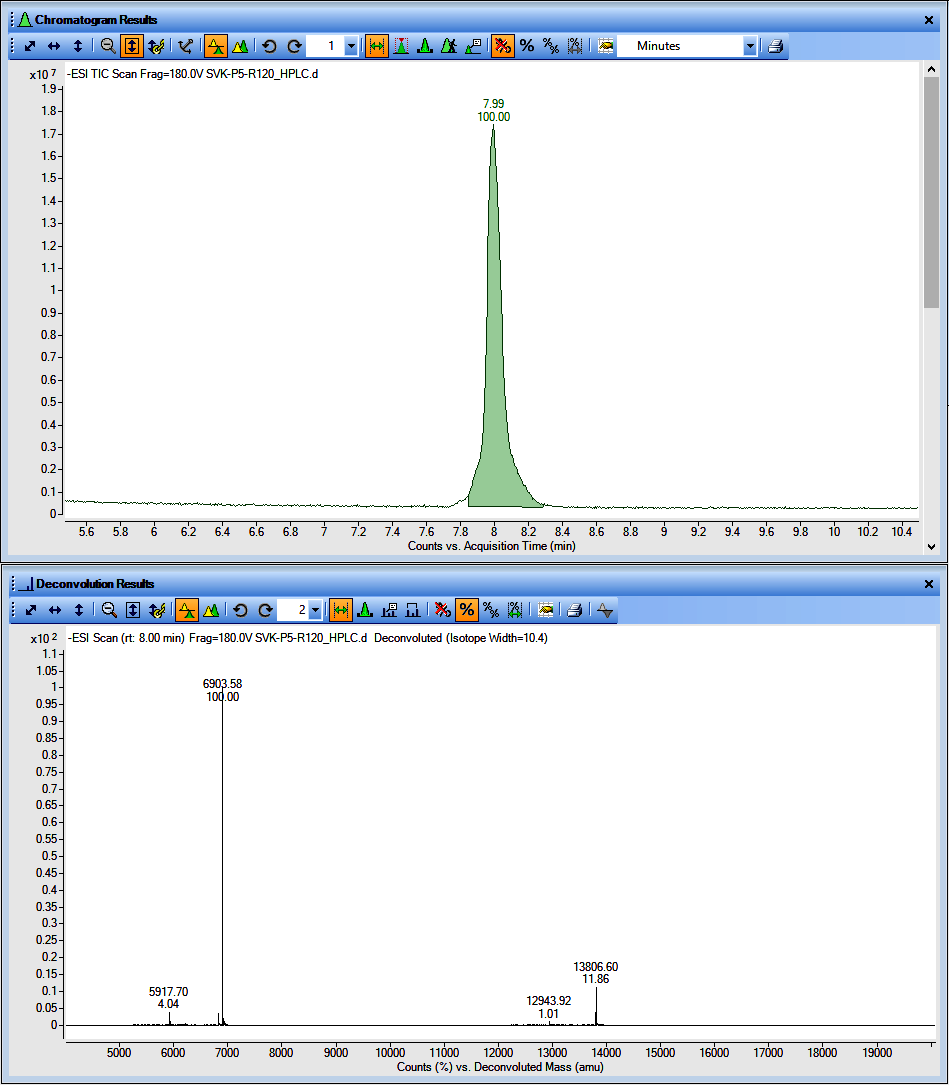


**Figure S3.** TIC and Deconvoluted mass spectrum of **1c**, expected: 6903; observed 6903.


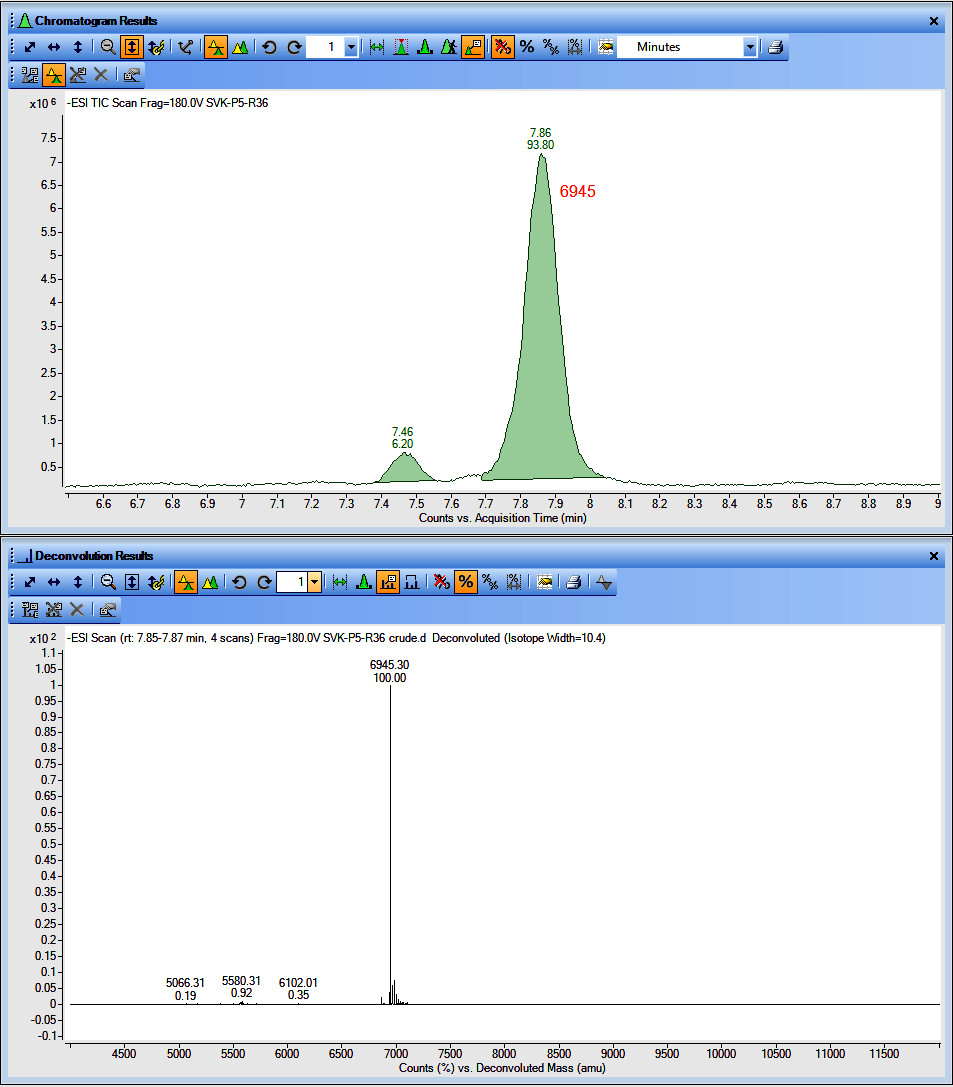

**Figure S4.** TIC and Deconvoluted mass spectrum of **3a**, expected: 6945; observed 6945.


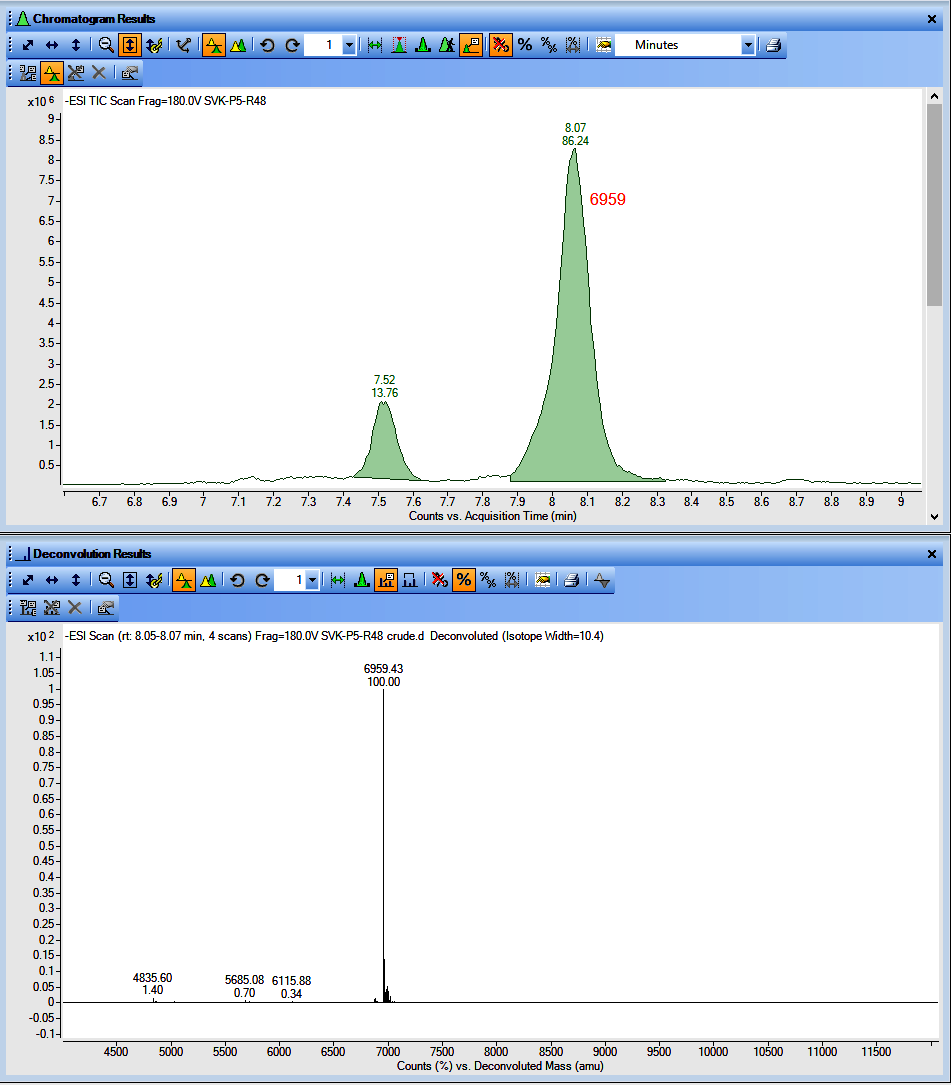

**Figure S5.** TIC and Deconvoluted mass spectrum of **3b**, expected: 6959; observed 6959.


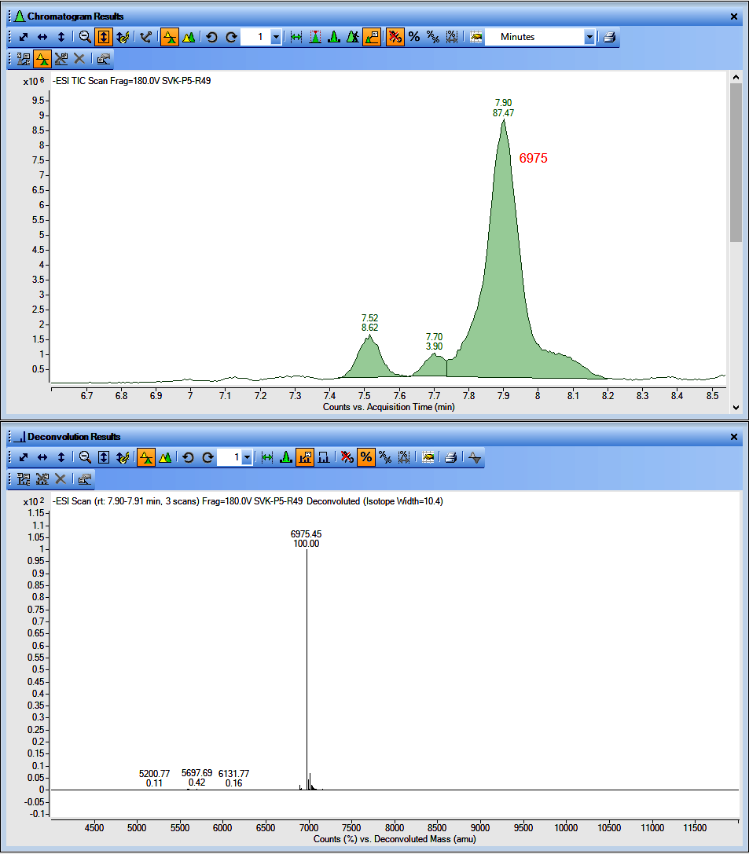

**Figure S6.** TIC and Deconvoluted mass spectrum of **3c**, expected: 6975; observed 6975.


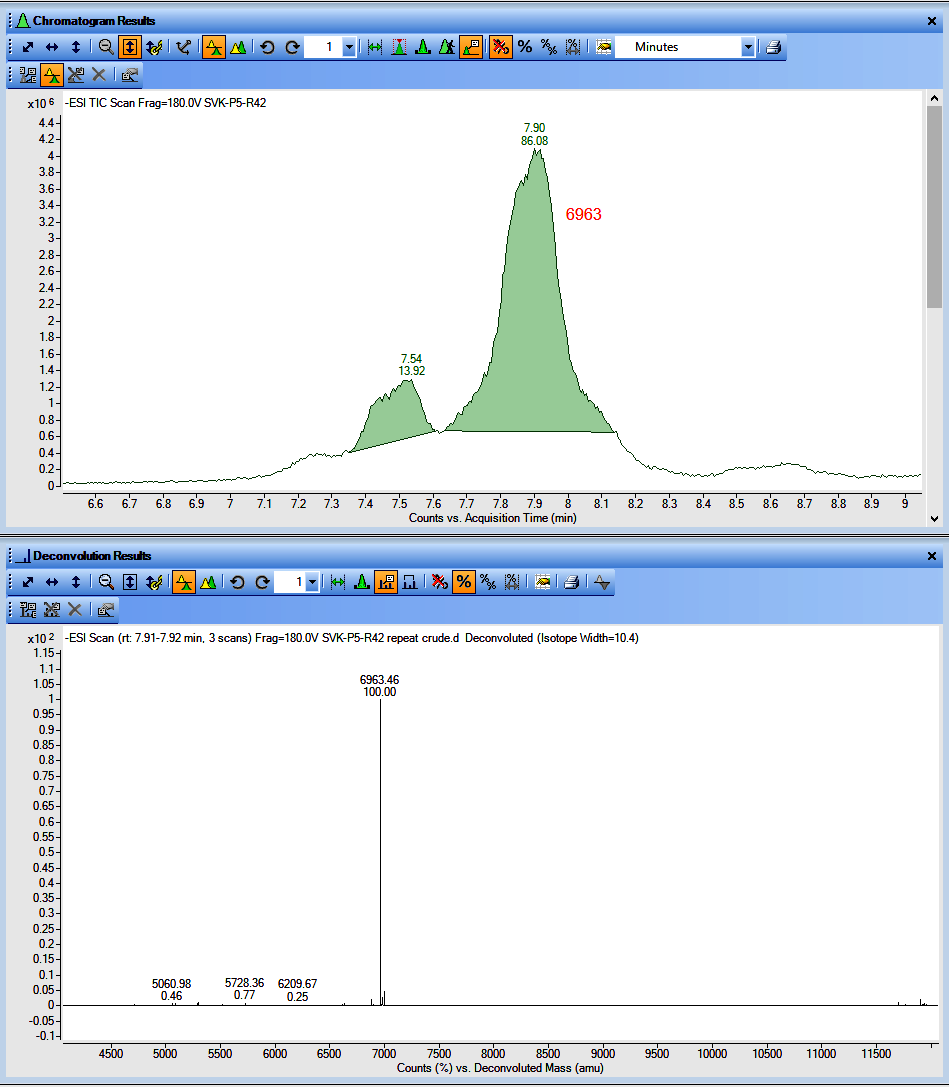

**Figure S7.** TIC and Deconvoluted mass spectrum of **3d**, expected: 6963; observed 6963.

**
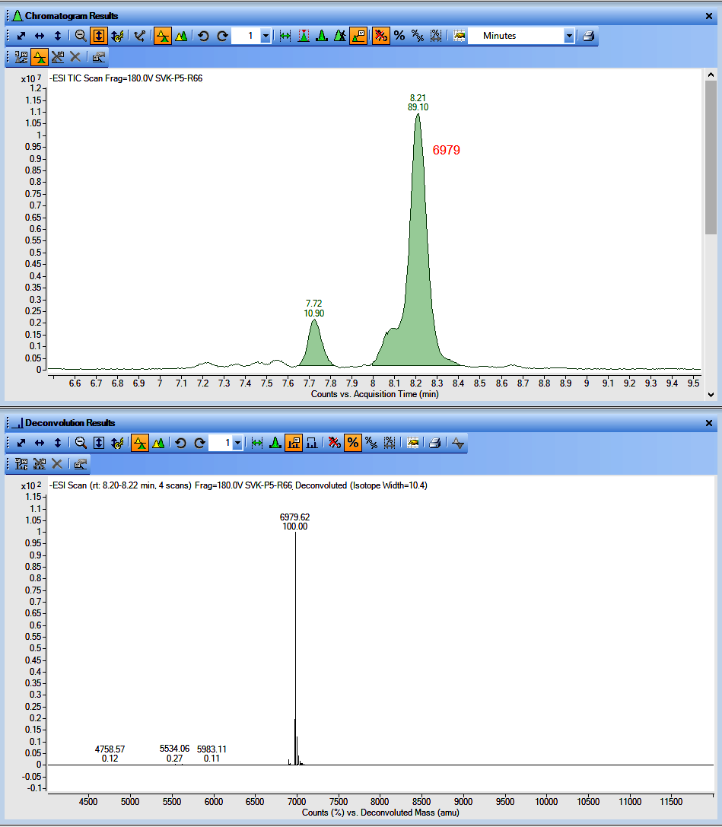
**

**Figure S8.** TIC and Deconvoluted mass spectrum of **3e**, expected: 6979; observed 6979.


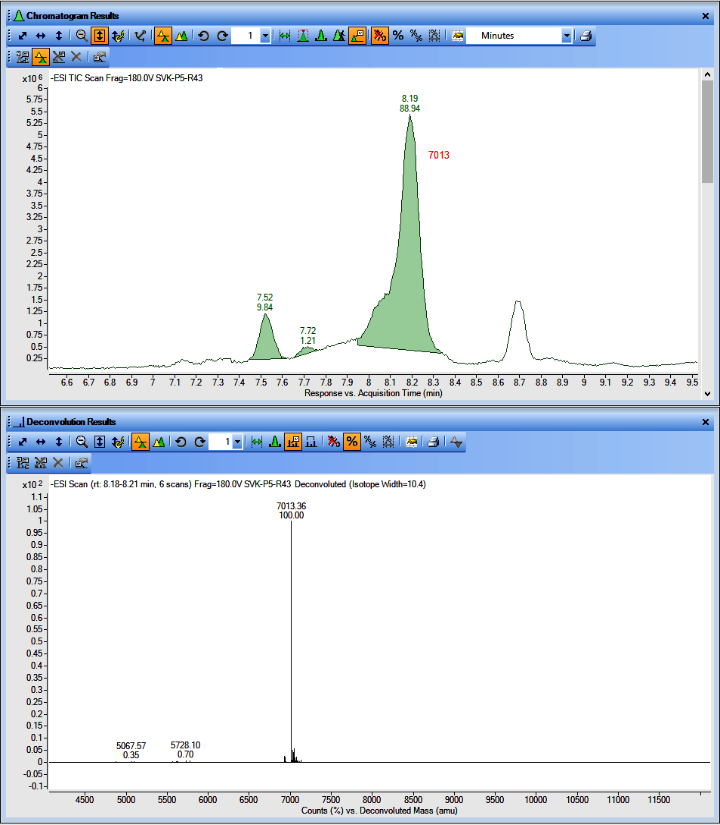

**Figure S9.** TIC and Deconvoluted mass spectrum of **3f**, expected: 7013; observed 7013.


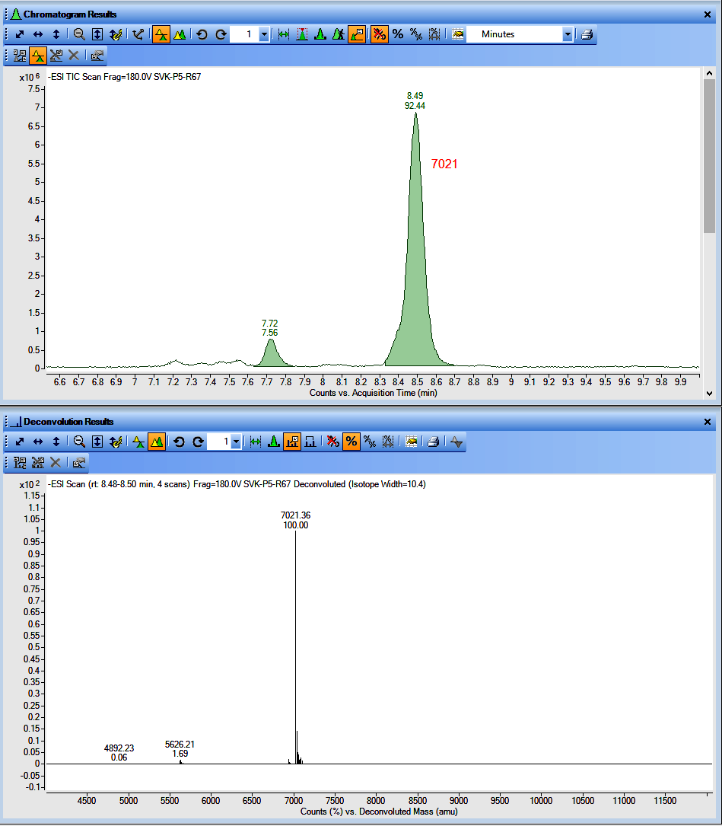

**Figure S10.** TIC and Deconvoluted mass spectrum of **3g**, expected: 7021; observed 7021.

**
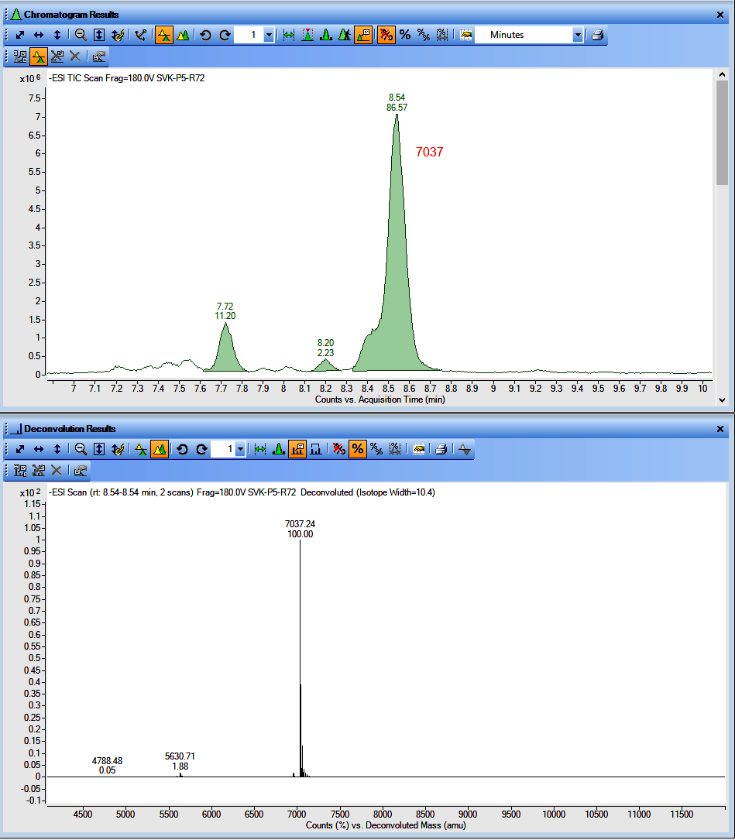
**

**Figure S11.** TIC and Deconvoluted mass spectrum of **3h**, expected: 7037; observed 7037.

**
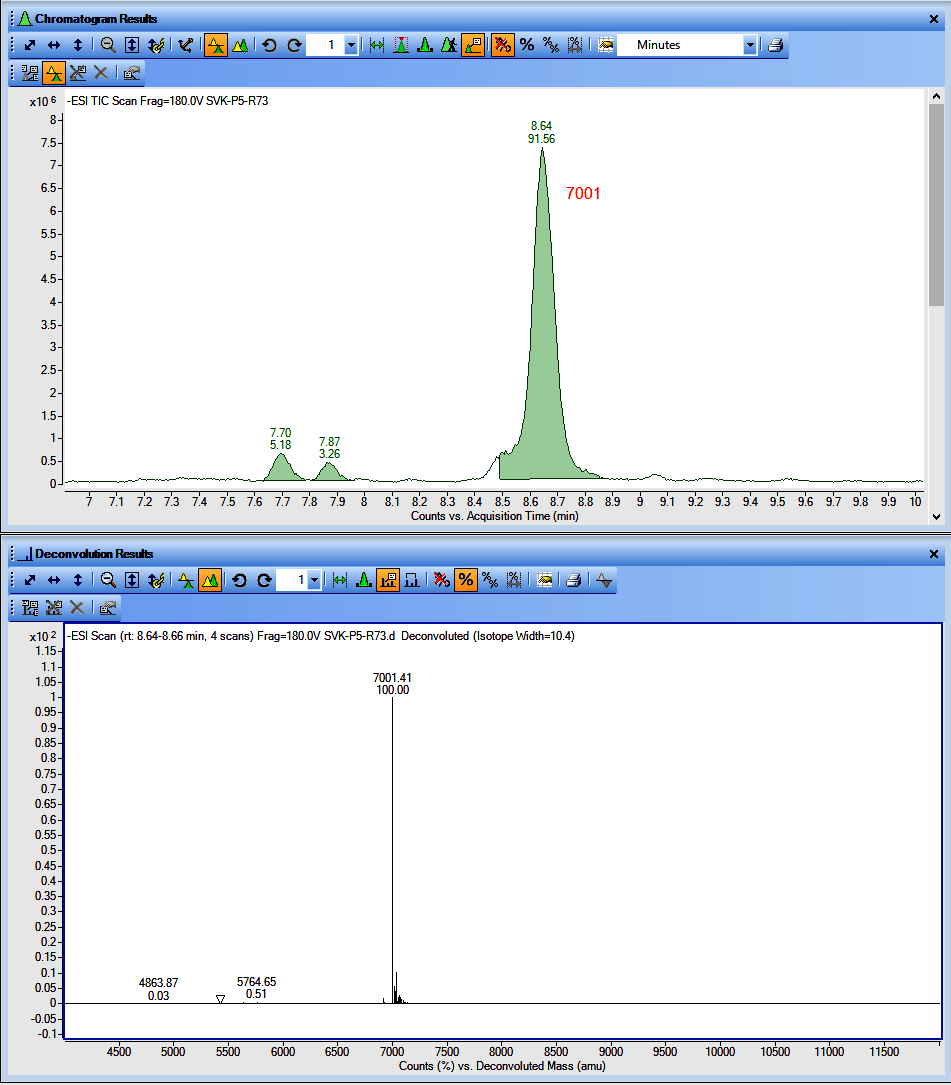
**

**Figure S12.** TIC and Deconvoluted mass spectrum of **3i**, expected: 7001; observed 7001.

**
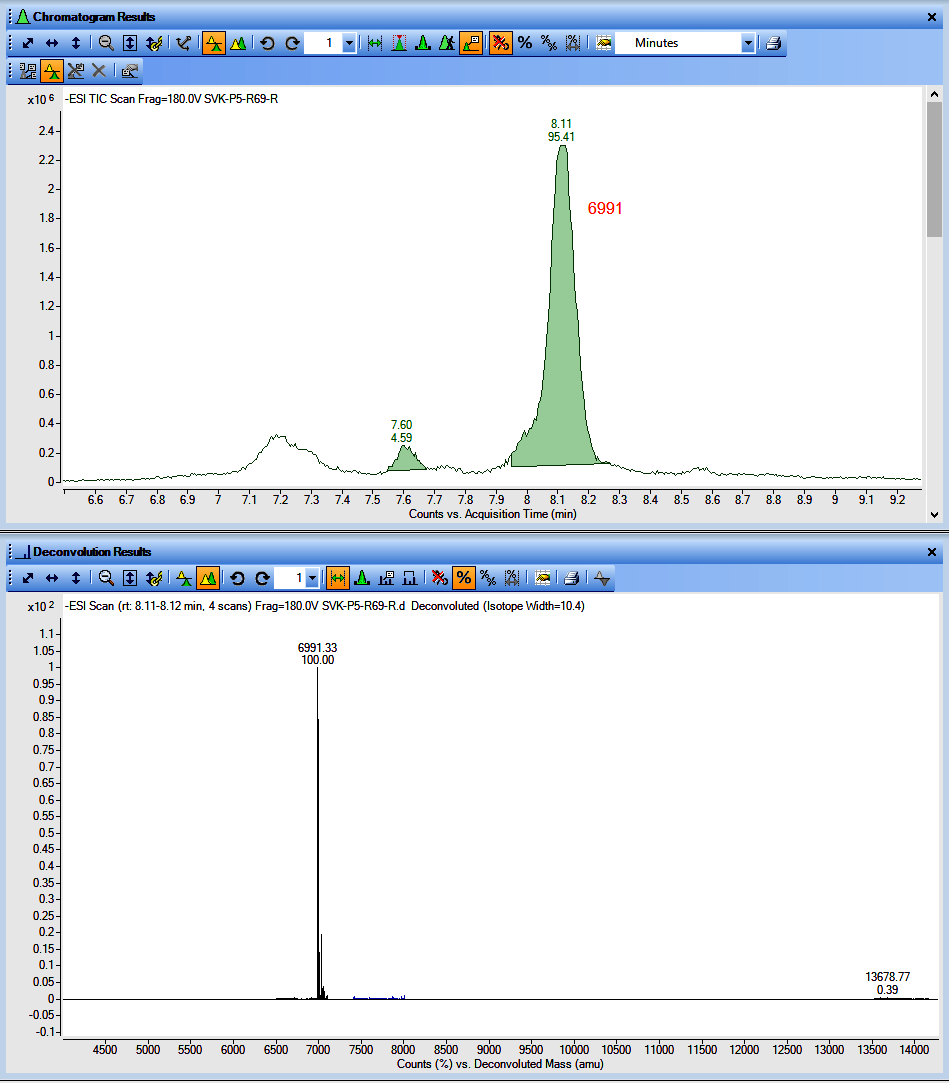
**

**Figure S13.** TIC and Deconvoluted mass spectrum of **3j**, expected: 6991; observed 6991.

**
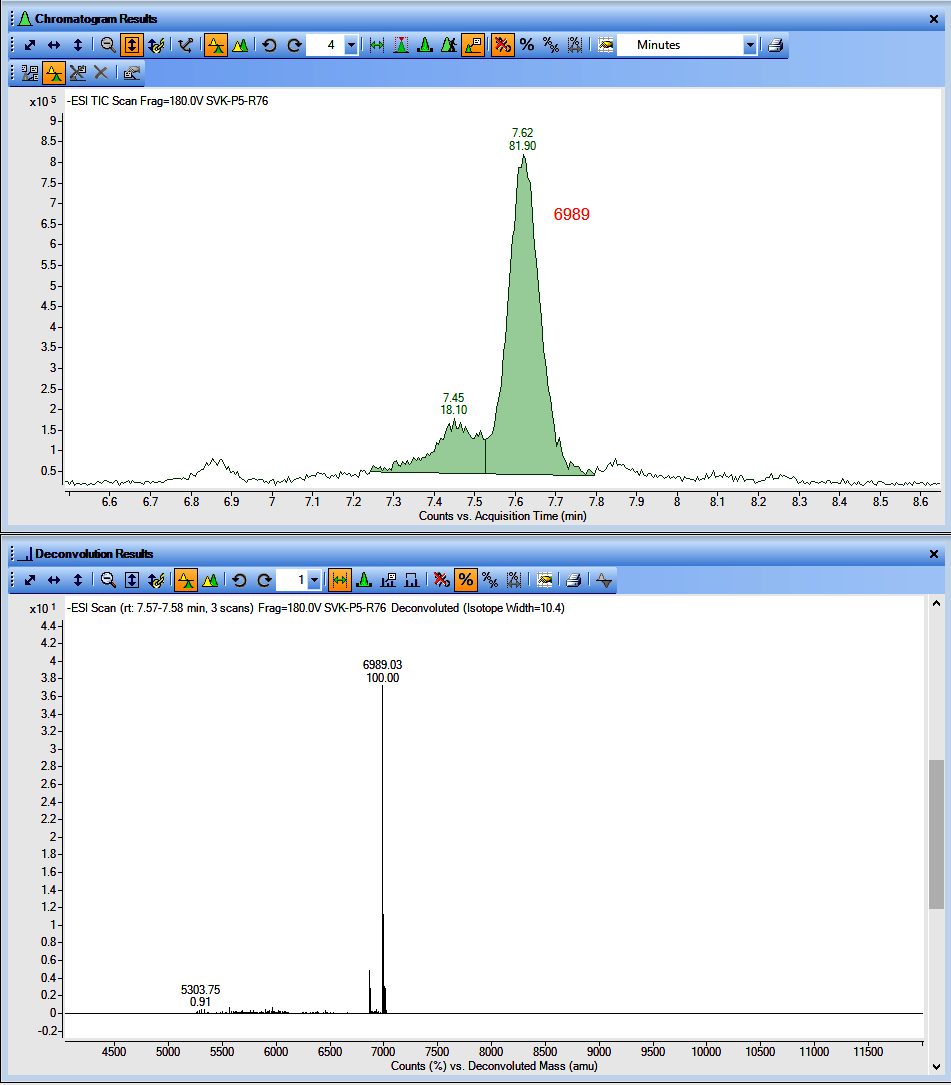
**

**Figure S14.** TIC and Deconvoluted mass spectrum of **3k**, expected: 6989; observed 6989.

**
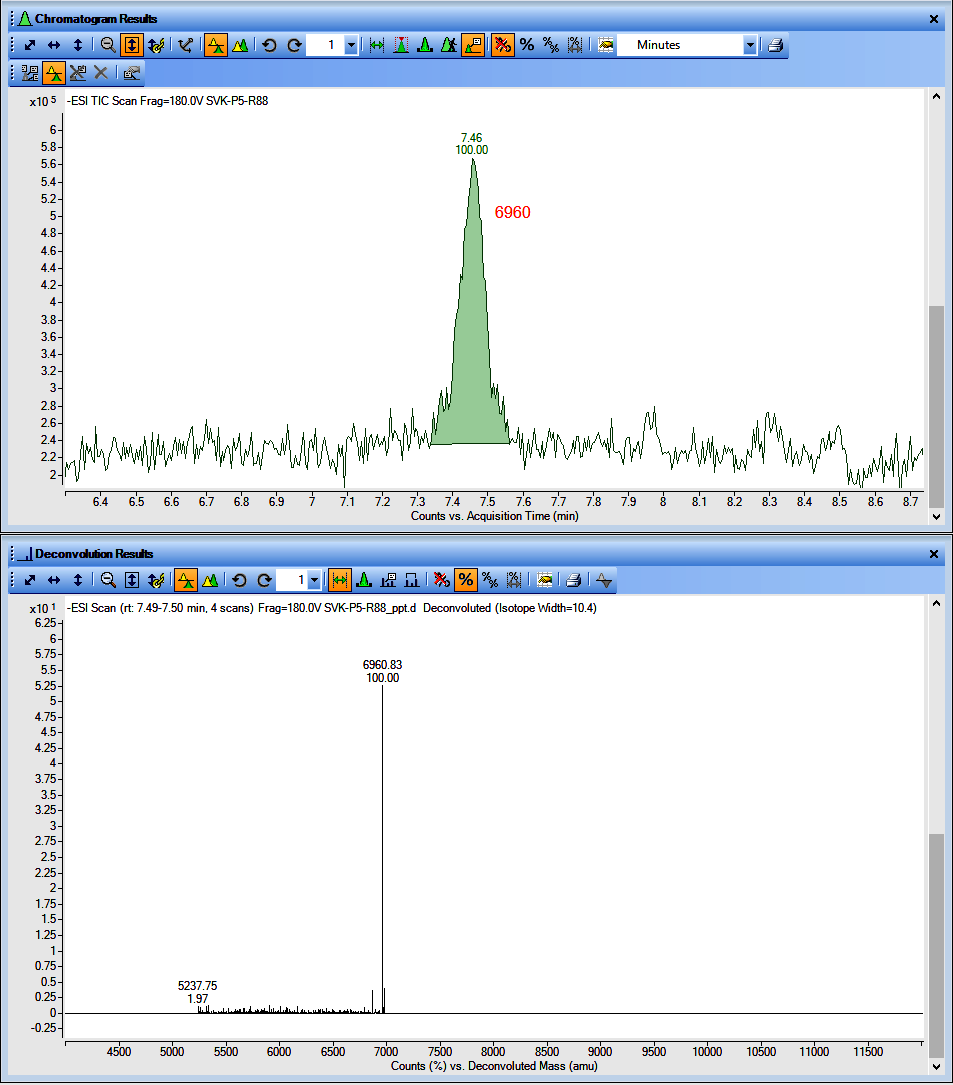
**

**Figure S15.** TIC and Deconvoluted mass spectrum of **3l**, expected: 6960; observed 6960.

**
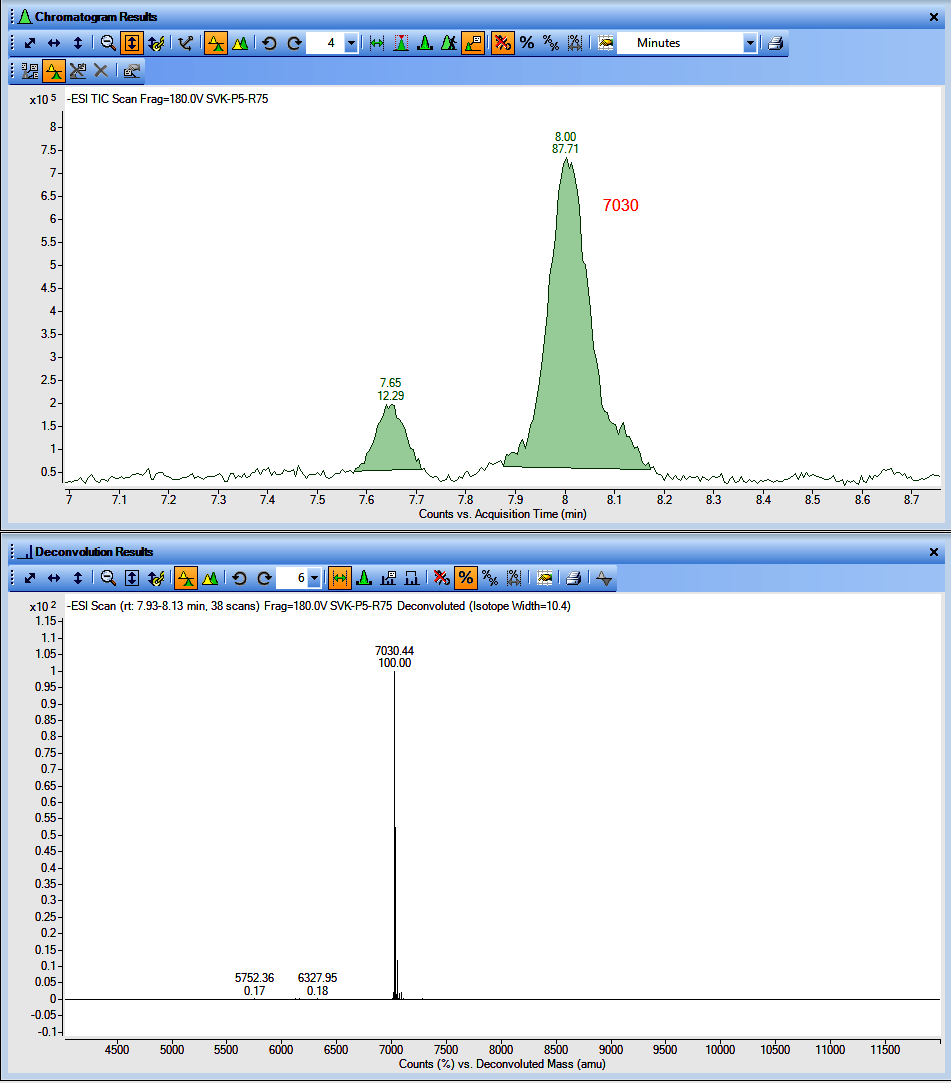
**

**Figure S16.** TIC and Deconvoluted mass spectrum of **3m**, expected: 7030; observed 7030.

**
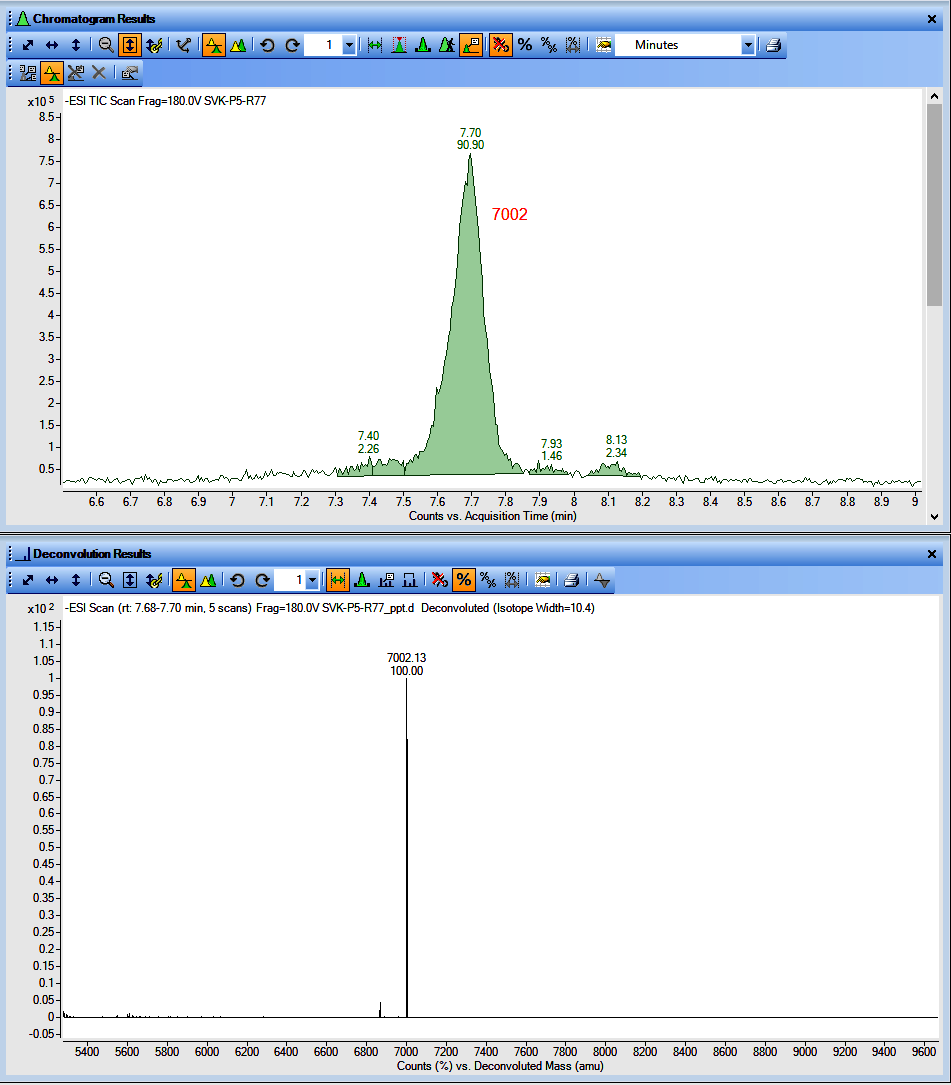
**

**Figure S17.** TIC and Deconvoluted mass spectrum of **3n**, expected: 7002; observed 7002.

**
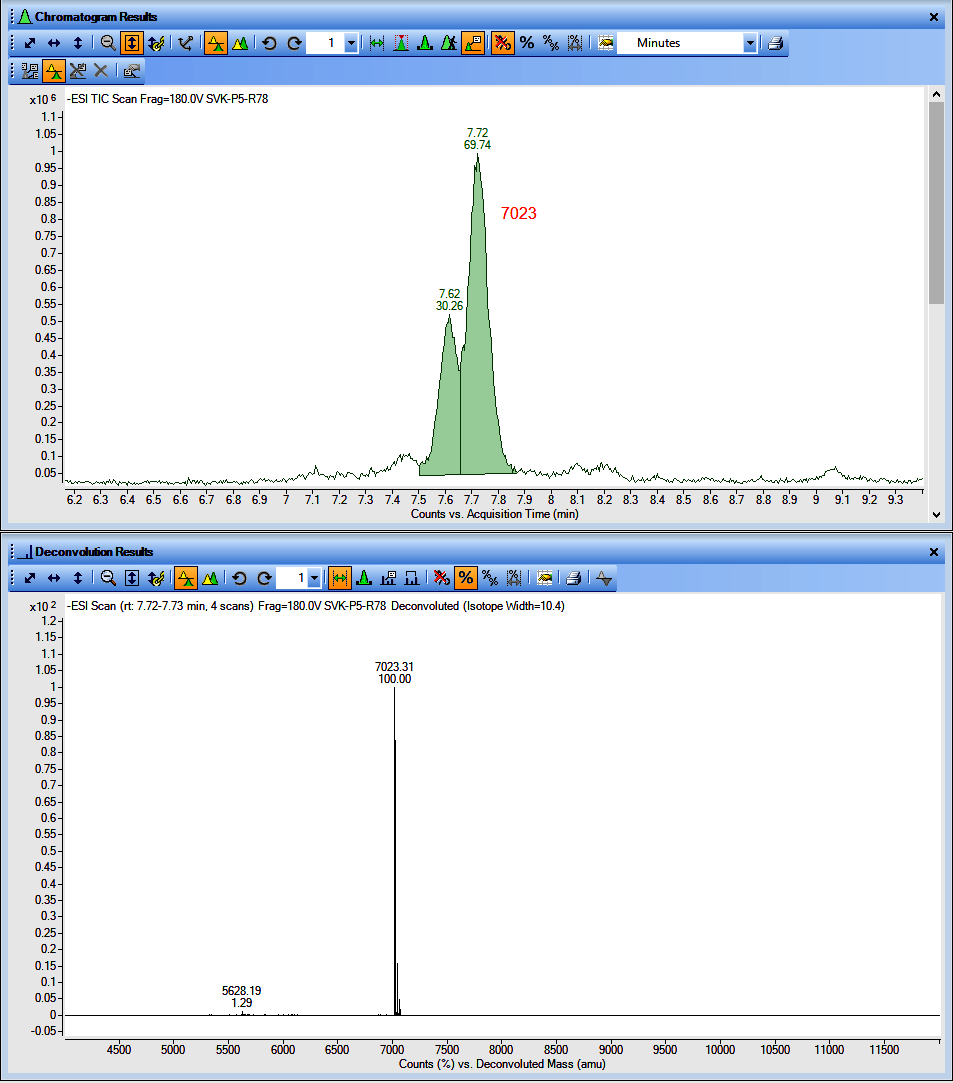
**

**Figure S18.** TIC and Deconvoluted mass spectrum of **3o**, expected: 7023; observed 7023.

**
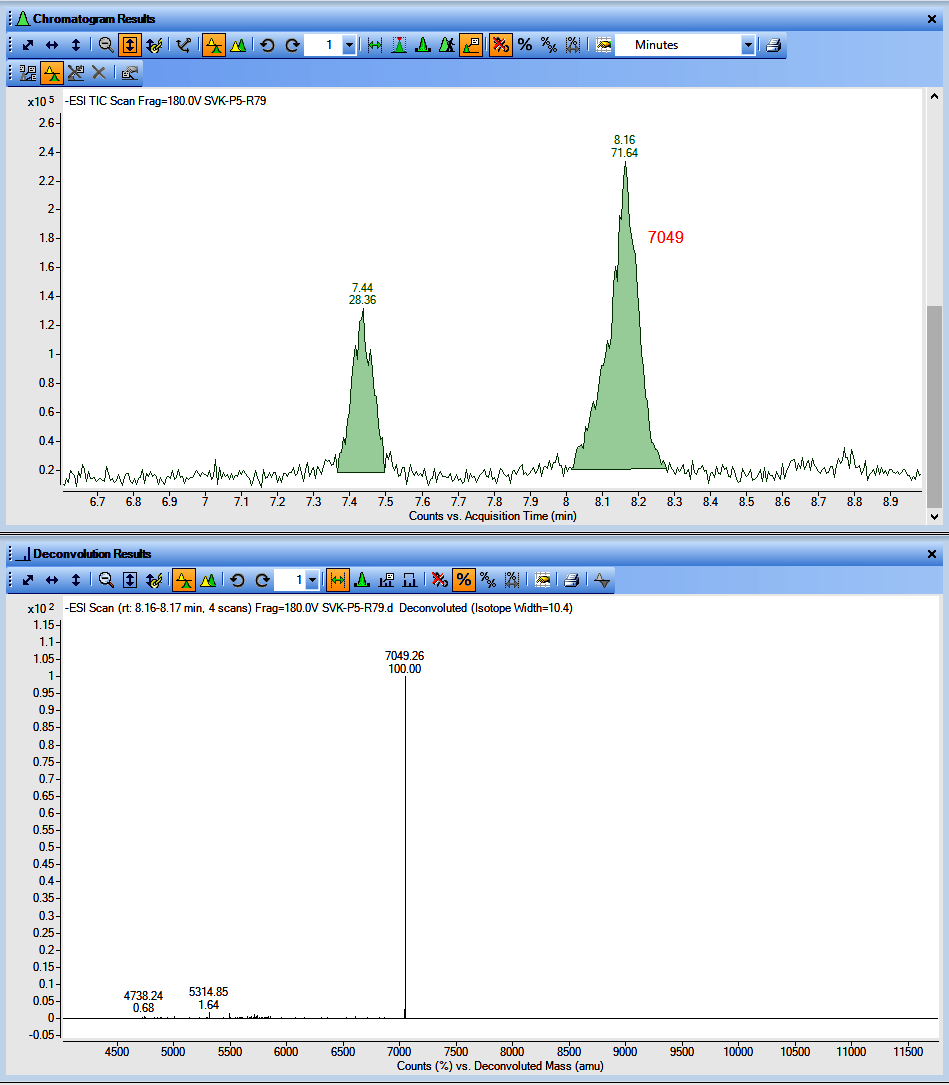
**

**Figure S19.** TIC and Deconvoluted mass spectrum of **3p**, expected: 7049; observed 7049.

**
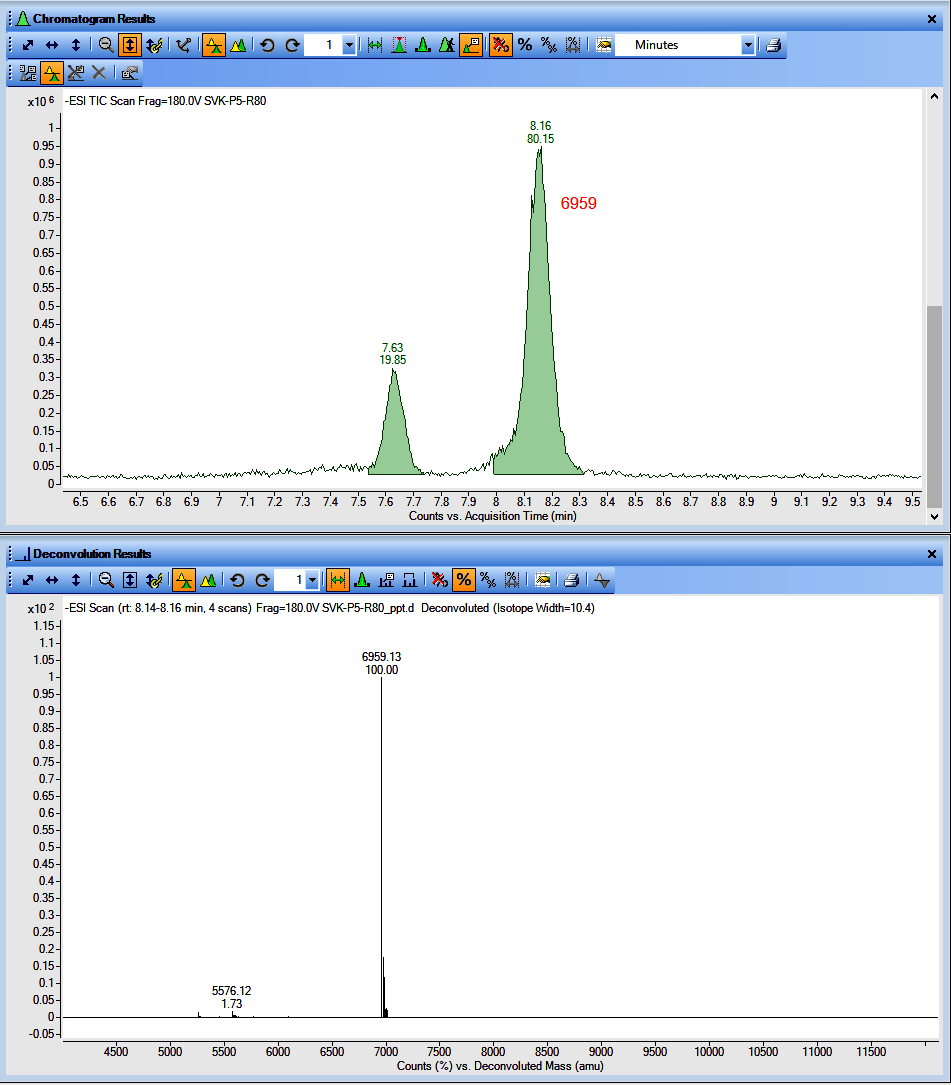
**

**Figure S20.** TIC and Deconvoluted mass spectrum of **3q**, expected: 6959; observed 6959.

**
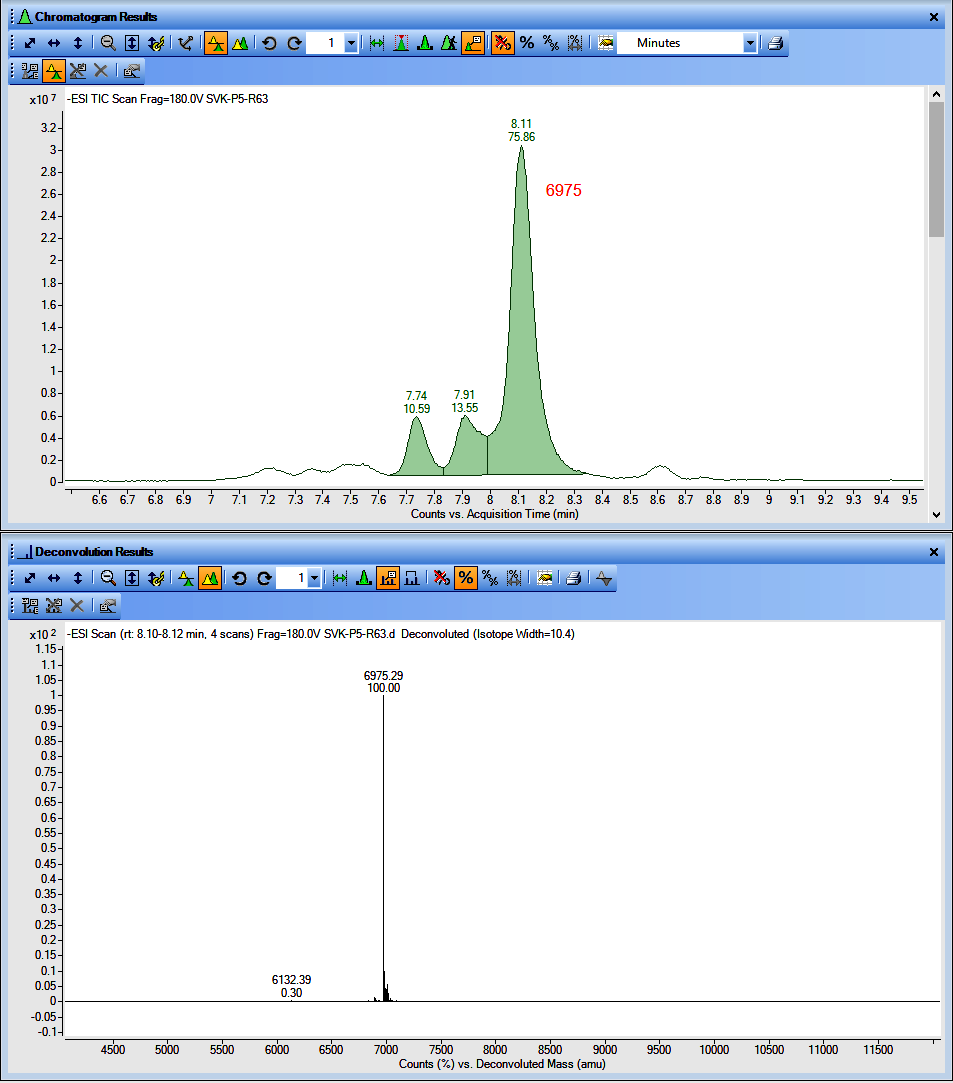
**

**Figure S21.** TIC and Deconvoluted mass spectrum of **3r**, expected: 6975; observed 6975.

**
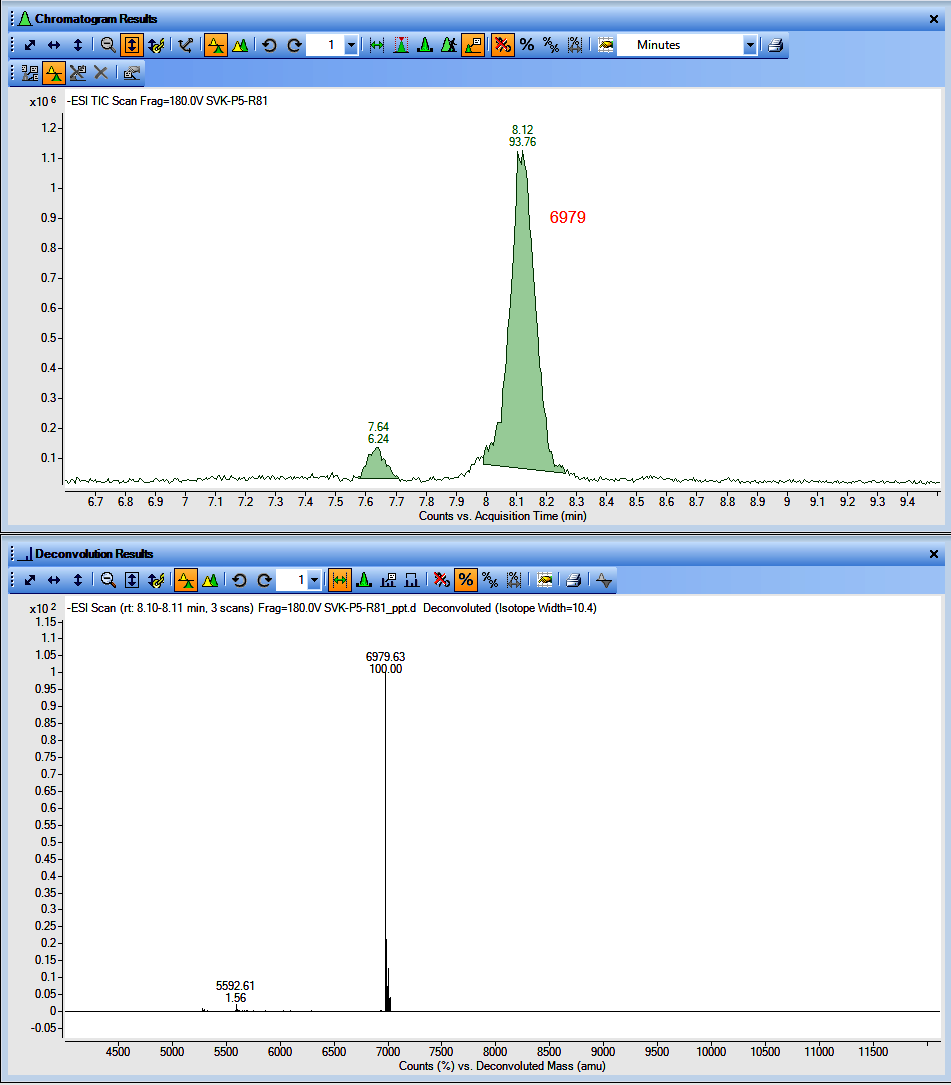
**

**Figure S22.** TIC and Deconvoluted mass spectrum of **3s**, expected: 6979; observed 6979.

**
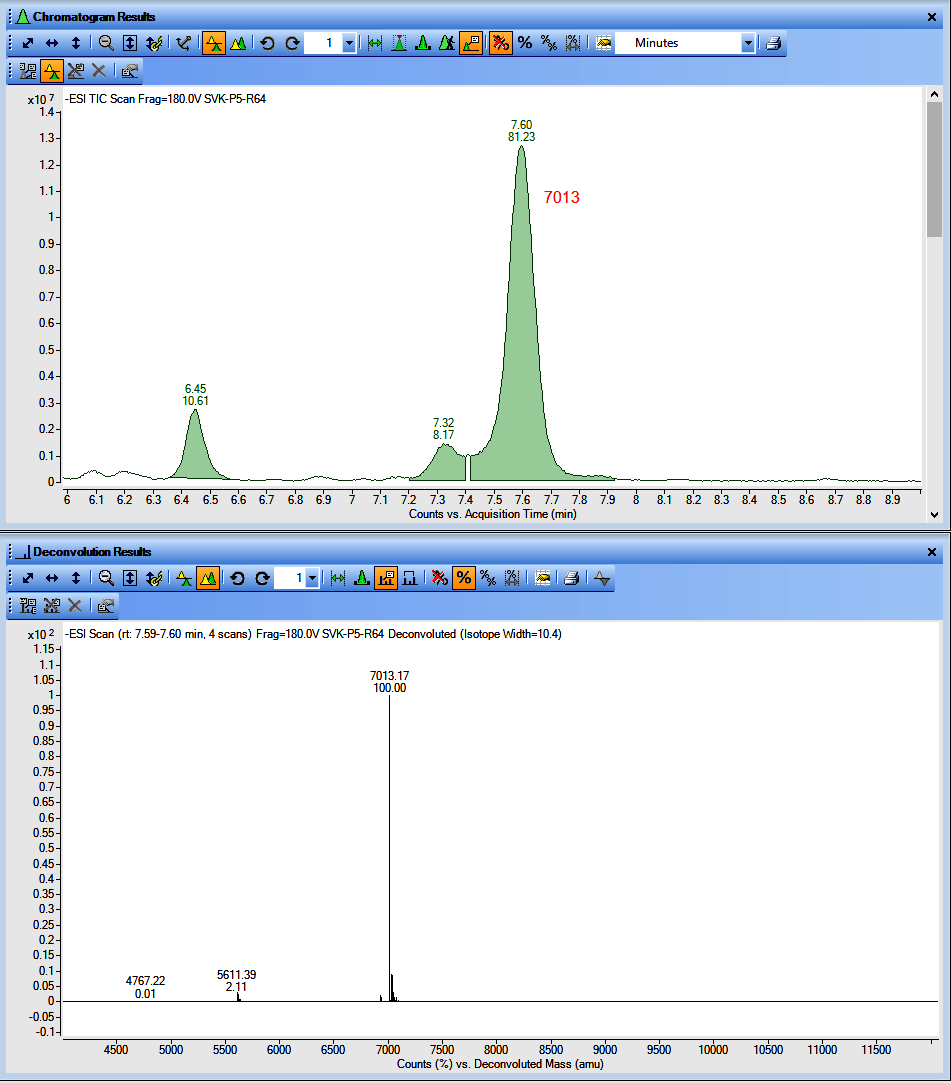
**

**Figure S23.** TIC and Deconvoluted mass spectrum of **3t**, expected: 7013; observed 7013.

**
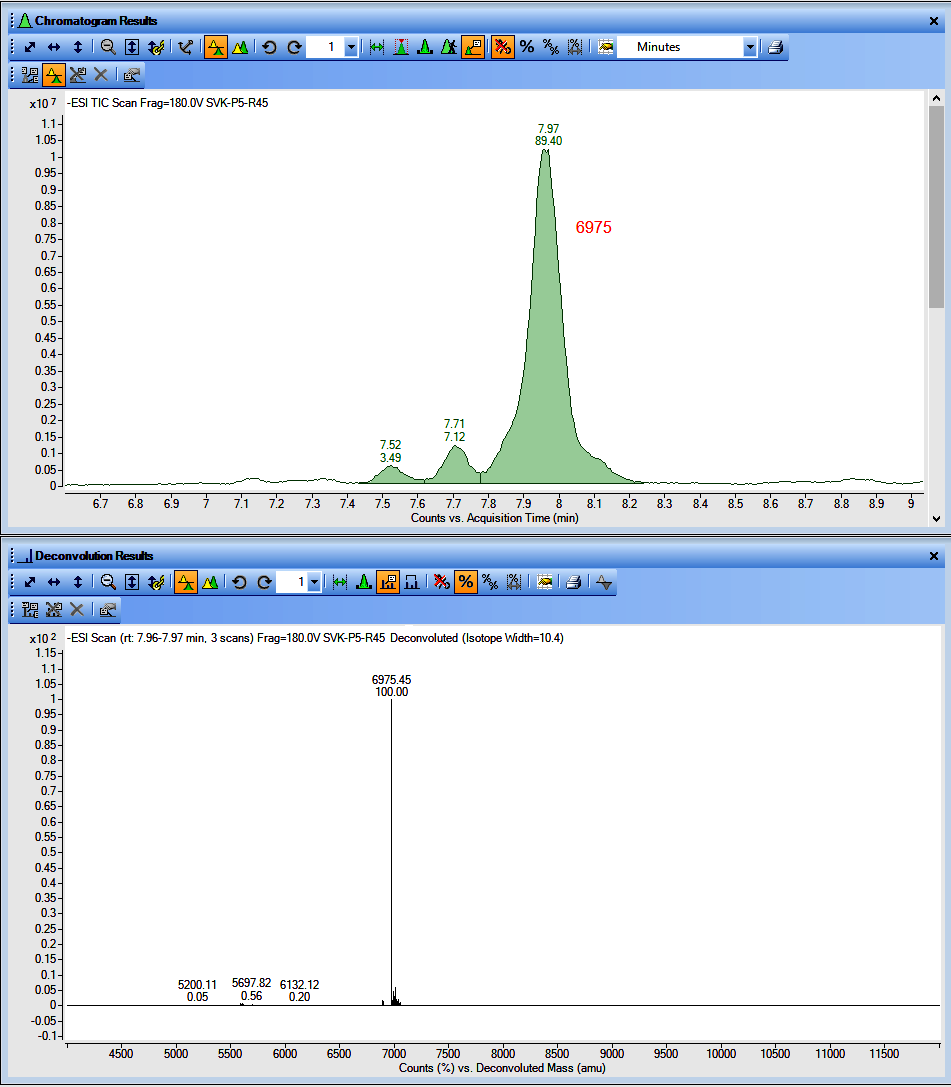
**

**Figure S24.** TIC and Deconvoluted mass spectrum of **3u**, expected: 6975; observed 6975.

**
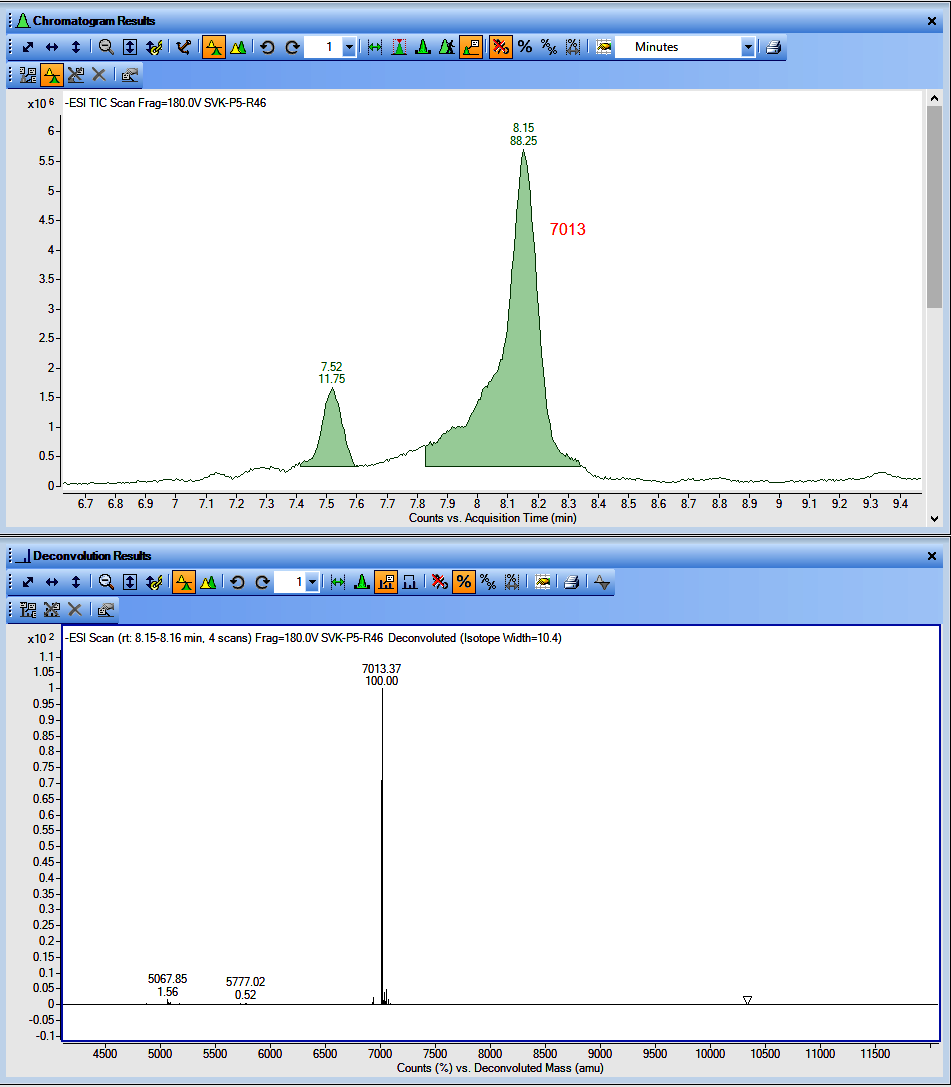
**

**Figure S25.** TIC and Deconvoluted mass spectrum of **3v**, expected: 7013; observed 7013.

**
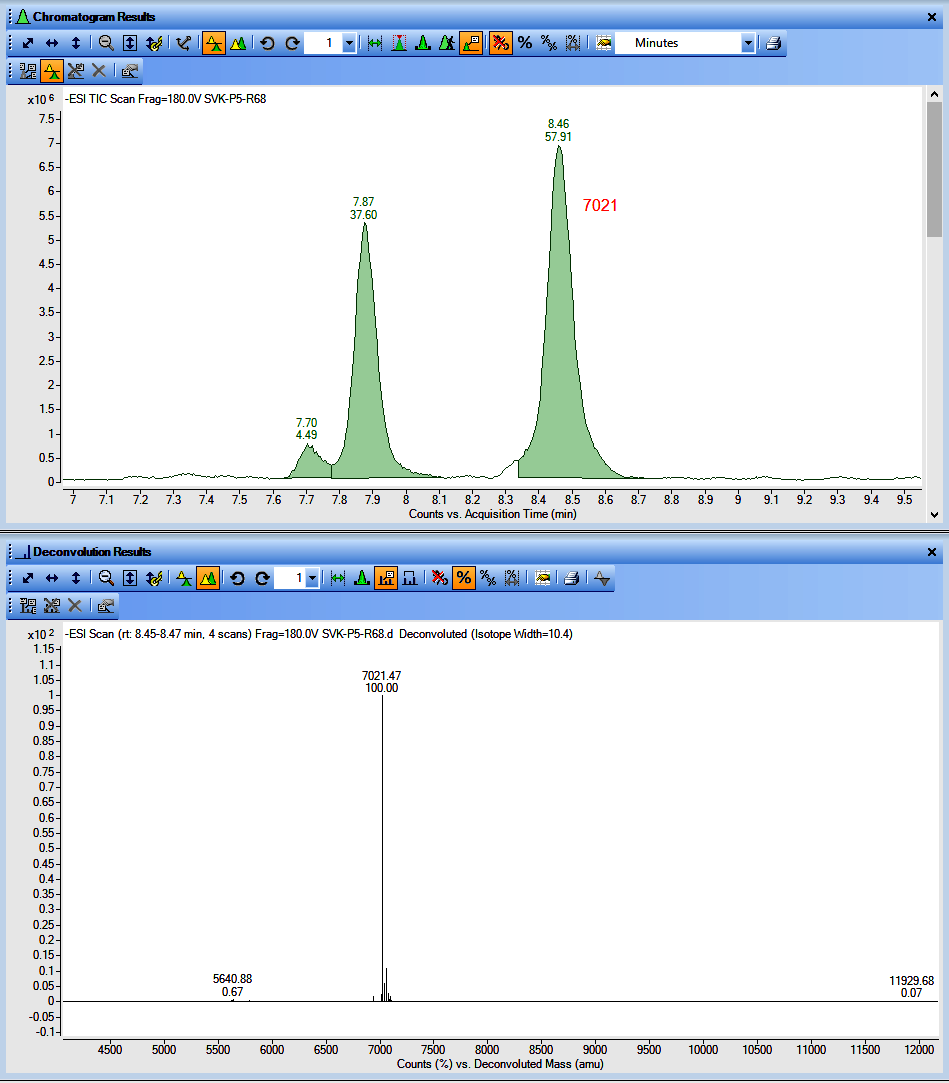
**

**Figure S26.** TIC and Deconvoluted mass spectrum of **3w**, expected: 7021; observed 7021.

**
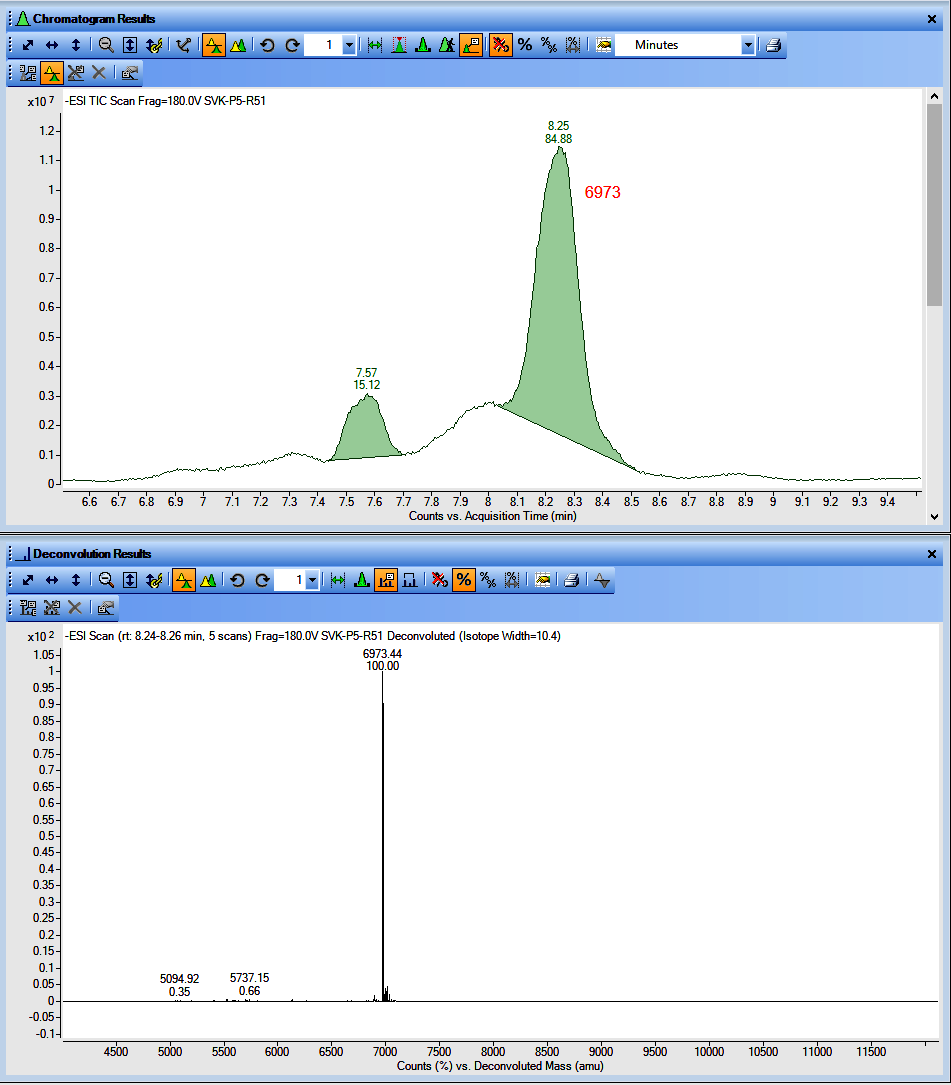
**

**Figure S27.** TIC and Deconvoluted mass spectrum of **3x**, expected: 6973; observed 6973.

**
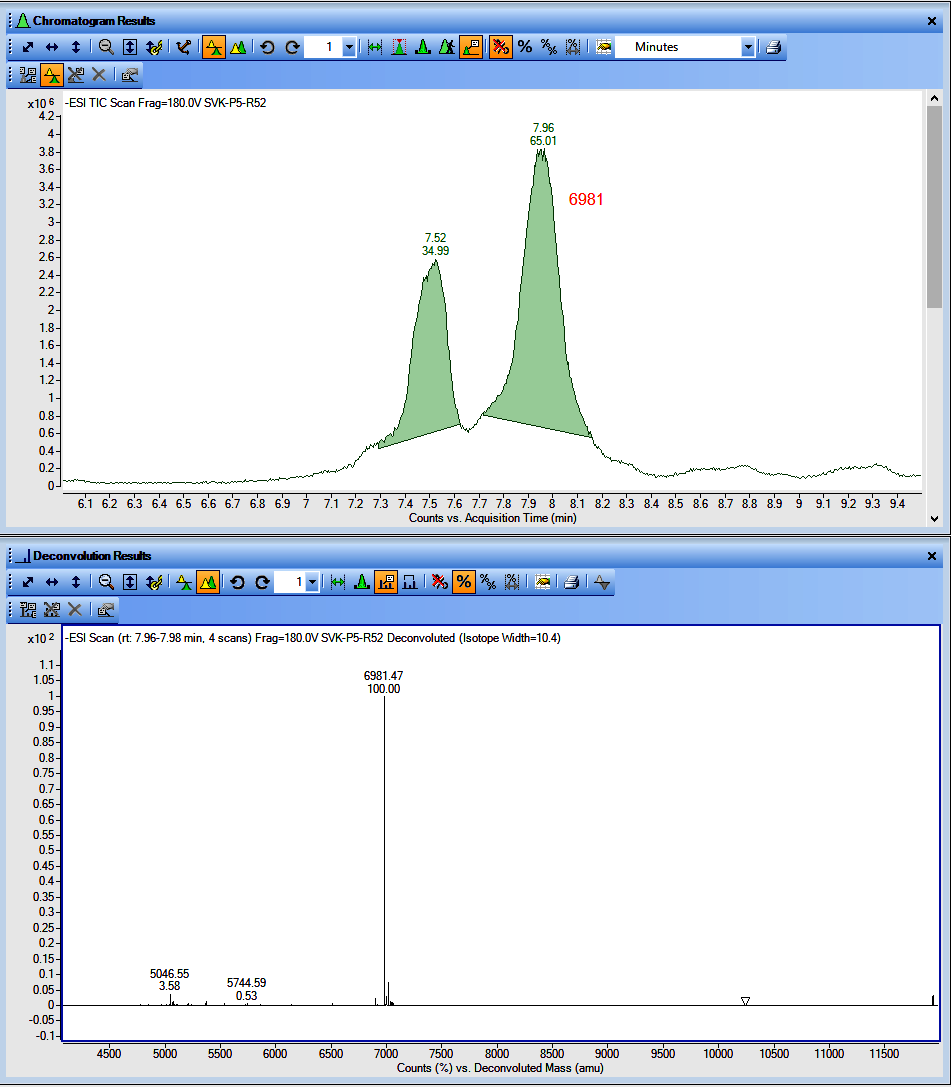
**

**Figure S28.** TIC and Deconvoluted mass spectrum of **3z**, expected: 6981; observed 6981.

**
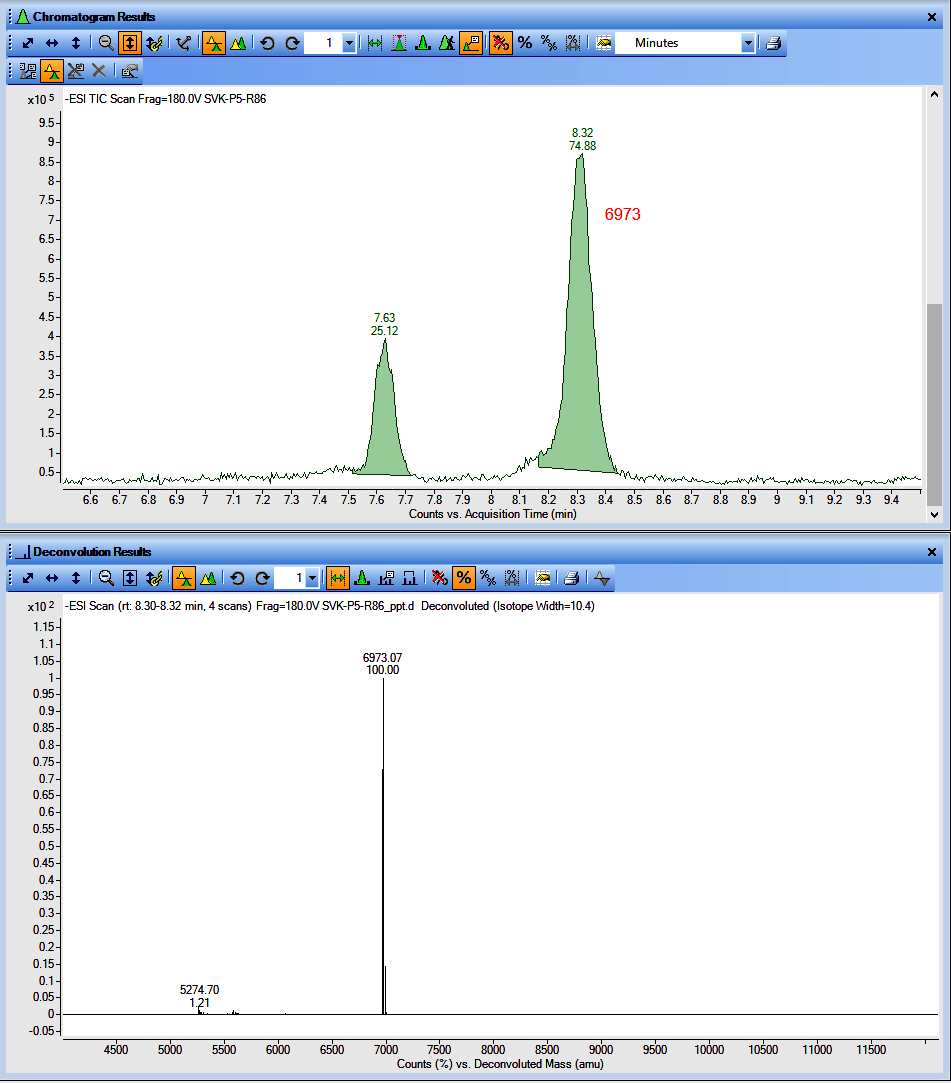
**

**Figure S29.** TIC and Deconvoluted mass spectrum of **3aa**, expected: 6973; observed 6973.

**
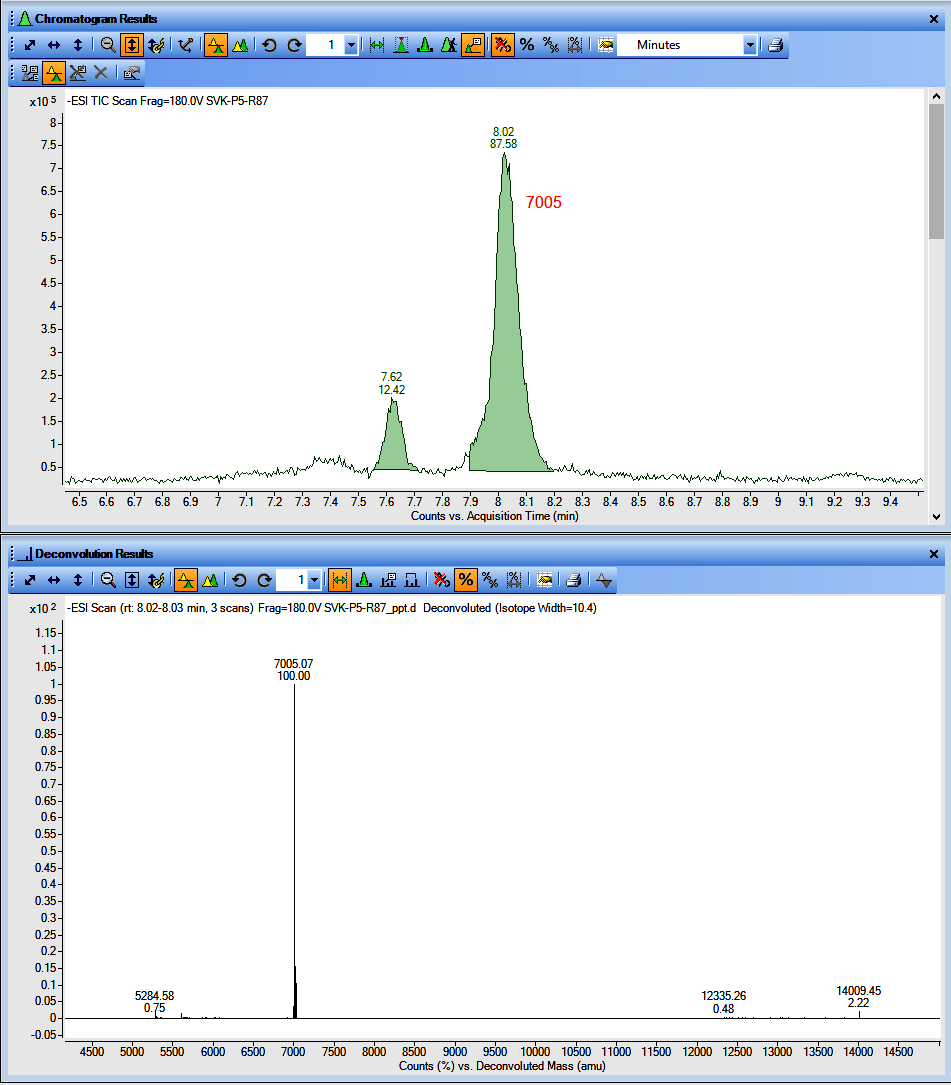
**

**Figure S30.** TIC and Deconvoluted mass spectrum of **3ab**, expected: 7005; observed 7005.

**
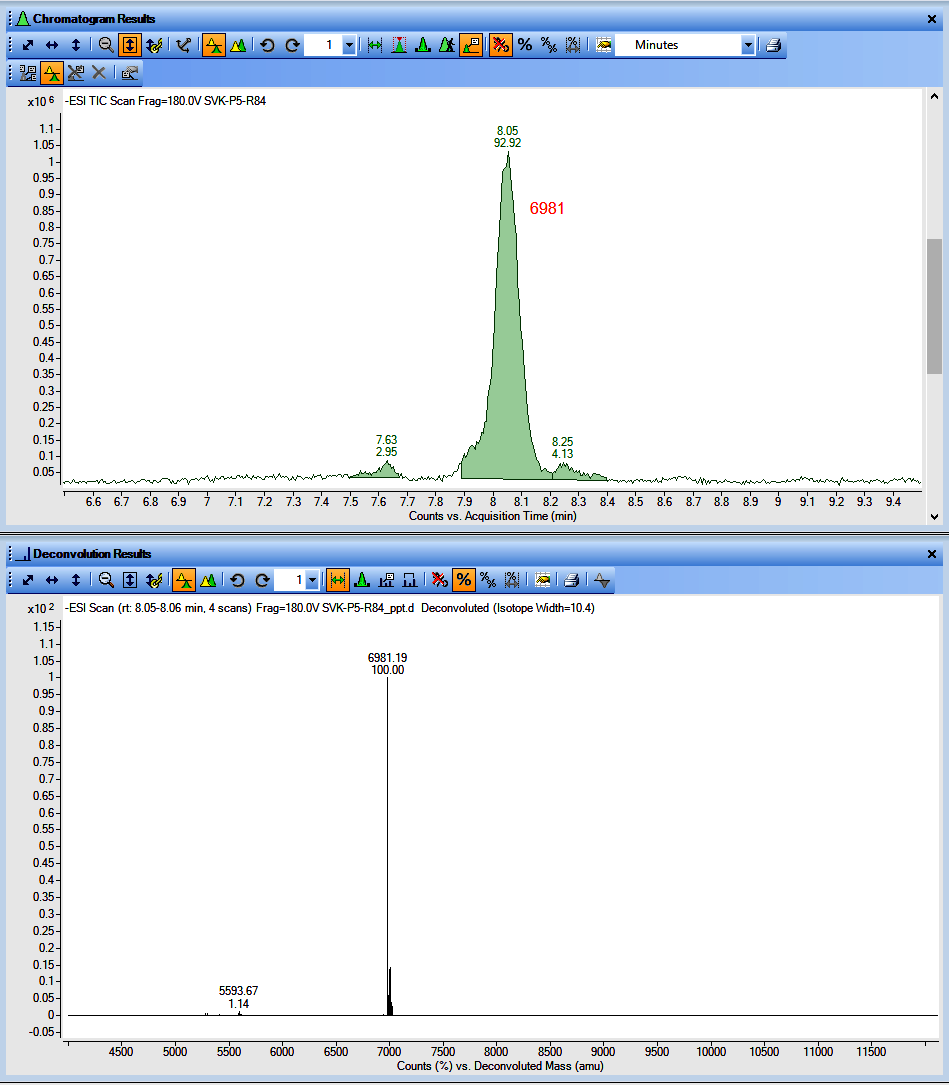
**

**Figure S31.** TIC and Deconvoluted mass spectrum of **3ac**, expected: 6981; observed 6981.

**
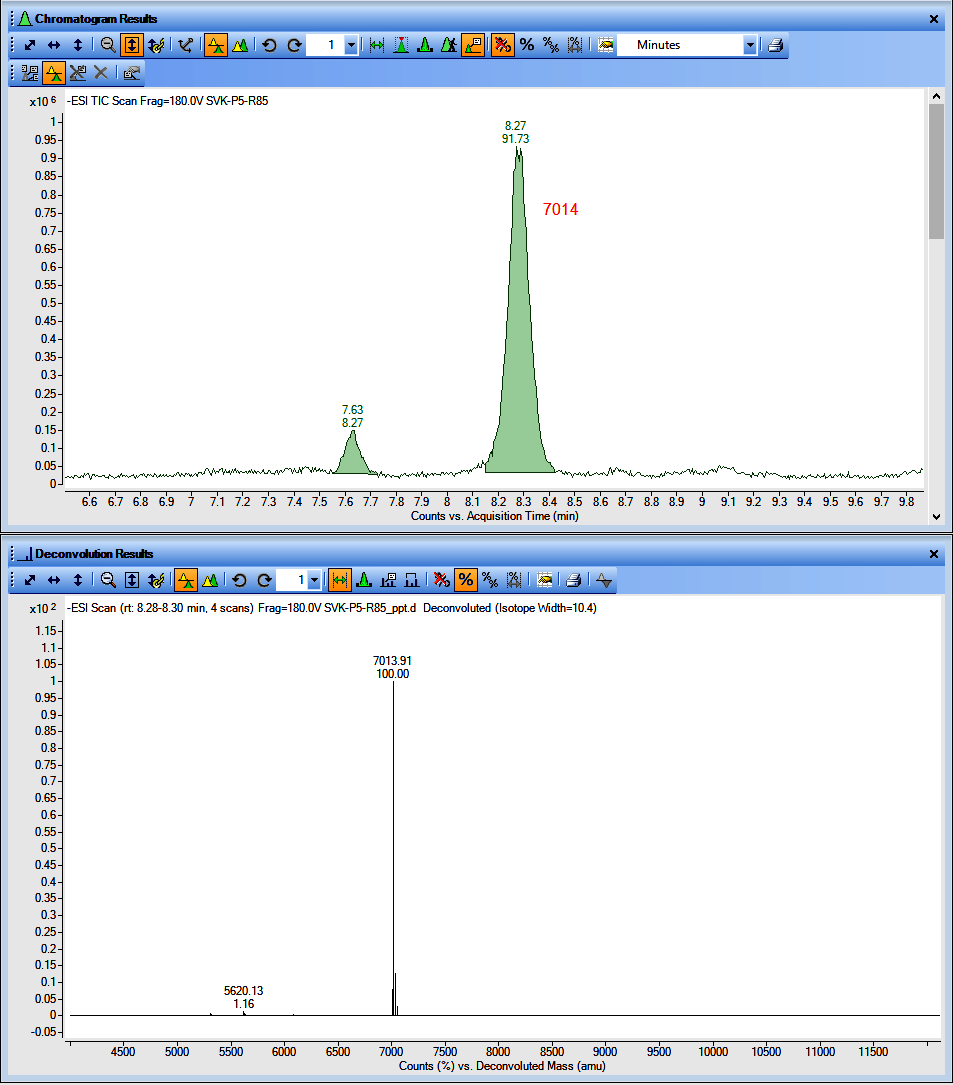
**

**Figure S32.** TIC and Deconvoluted mass spectrum of **3ad**, expected: 7014; observed 7014.

**
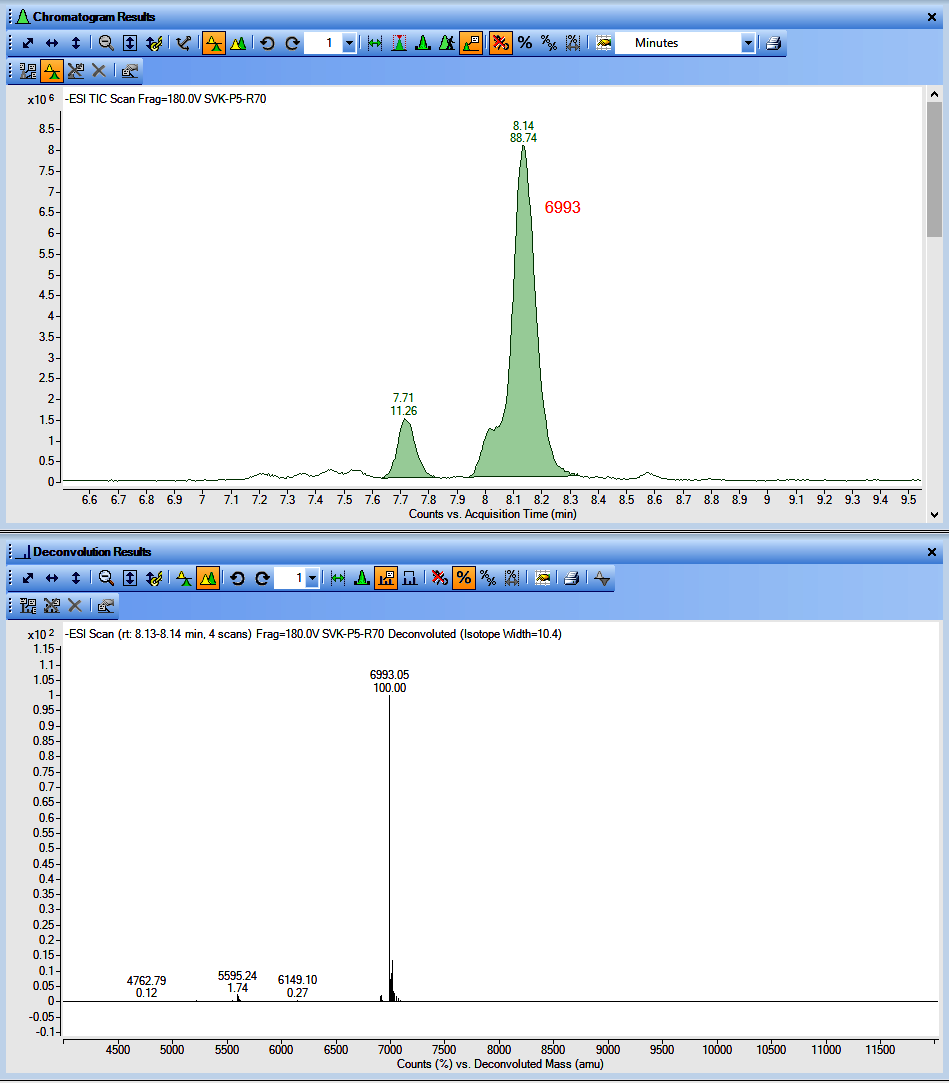
**

**Figure S33.** TIC and Deconvoluted mass spectrum of **3ae**, expected: 6993; observed 6993.

**
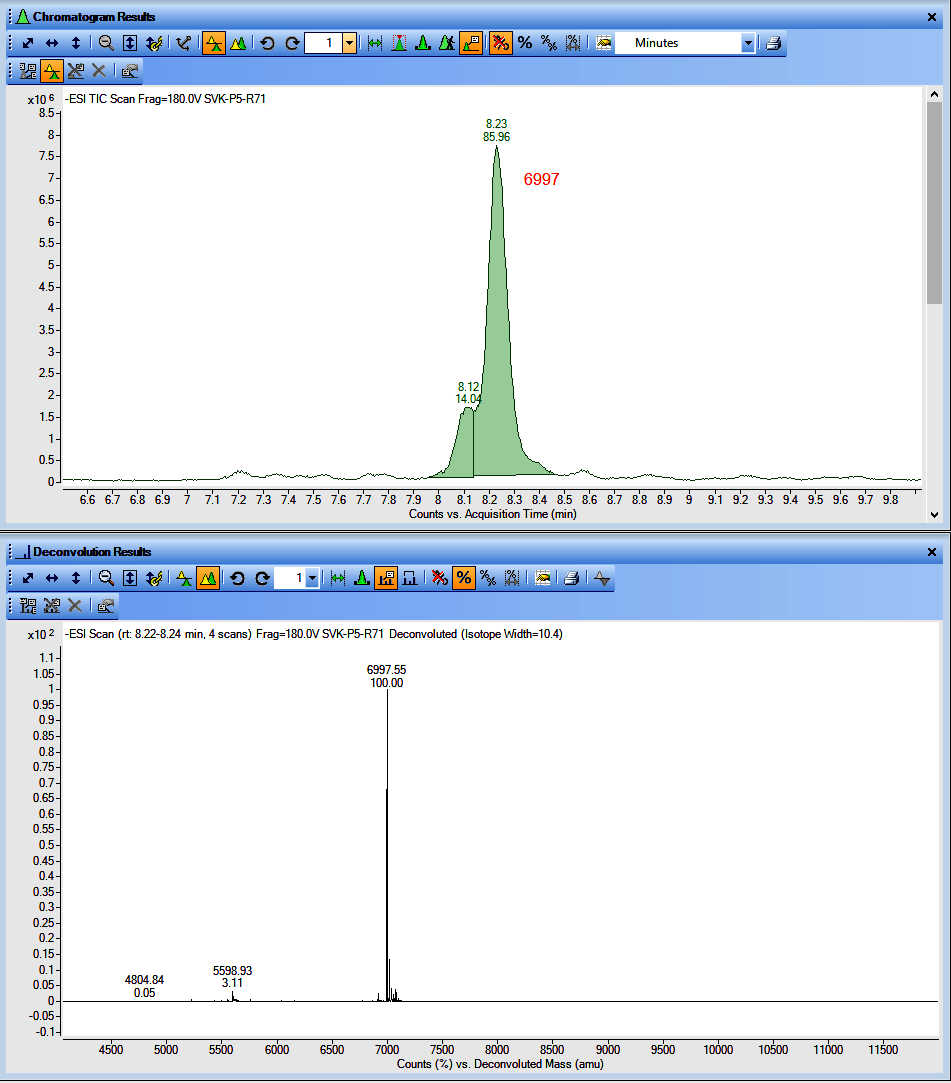
**

**Figure S34.** TIC and Deconvoluted mass spectrum of **3af**, expected: 6997; observed 6997.

**
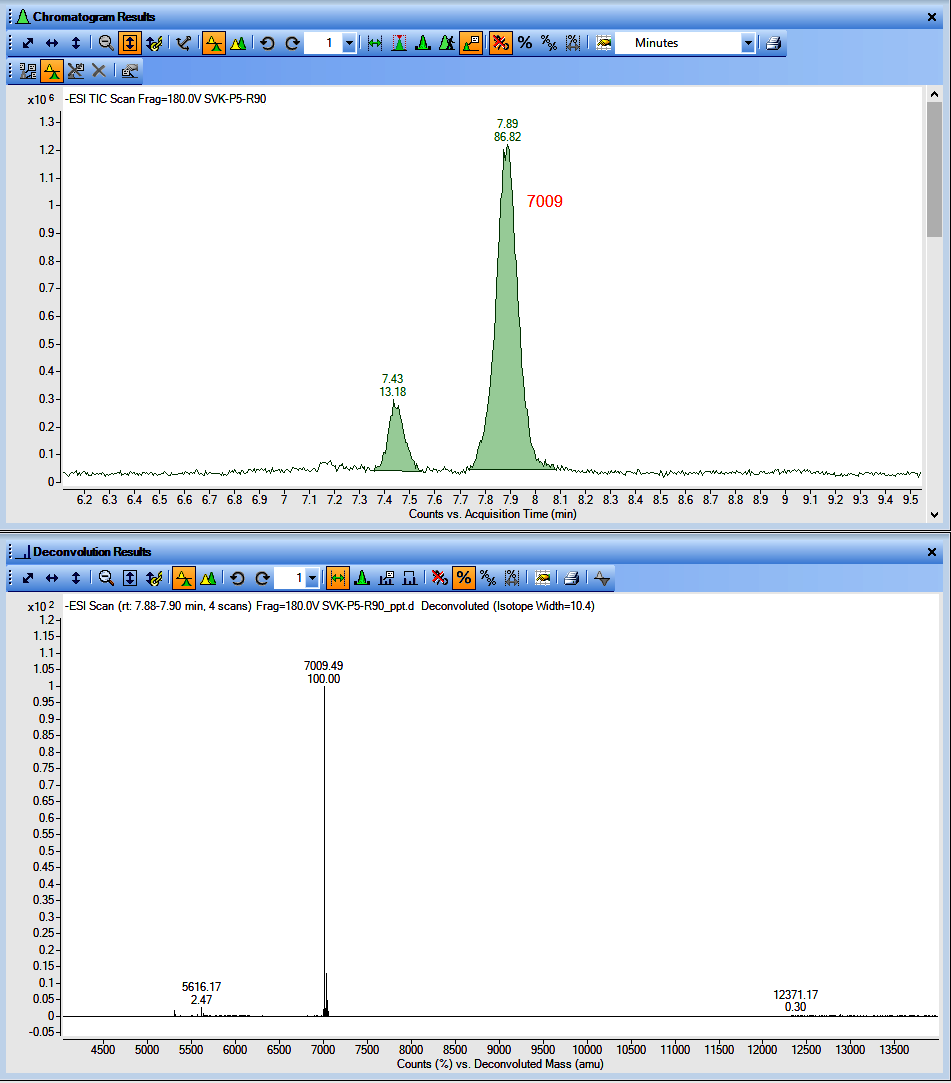
**

**Figure S35.** TIC and Deconvoluted mass spectrum of **3ag**, expected: 7009; observed 7009.

**
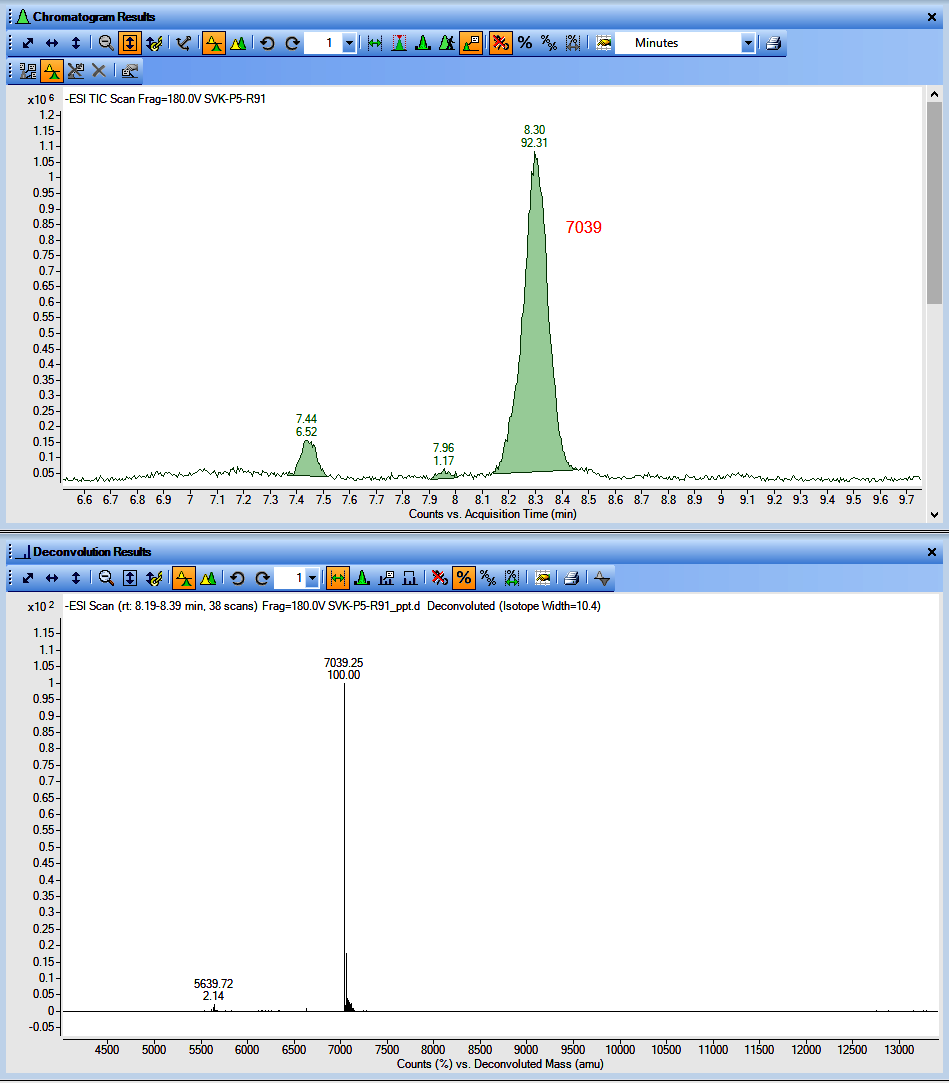
**

**Figure S36.** TIC and Deconvoluted mass spectrum of **3ah**, expected: 7039; observed 7039.

**
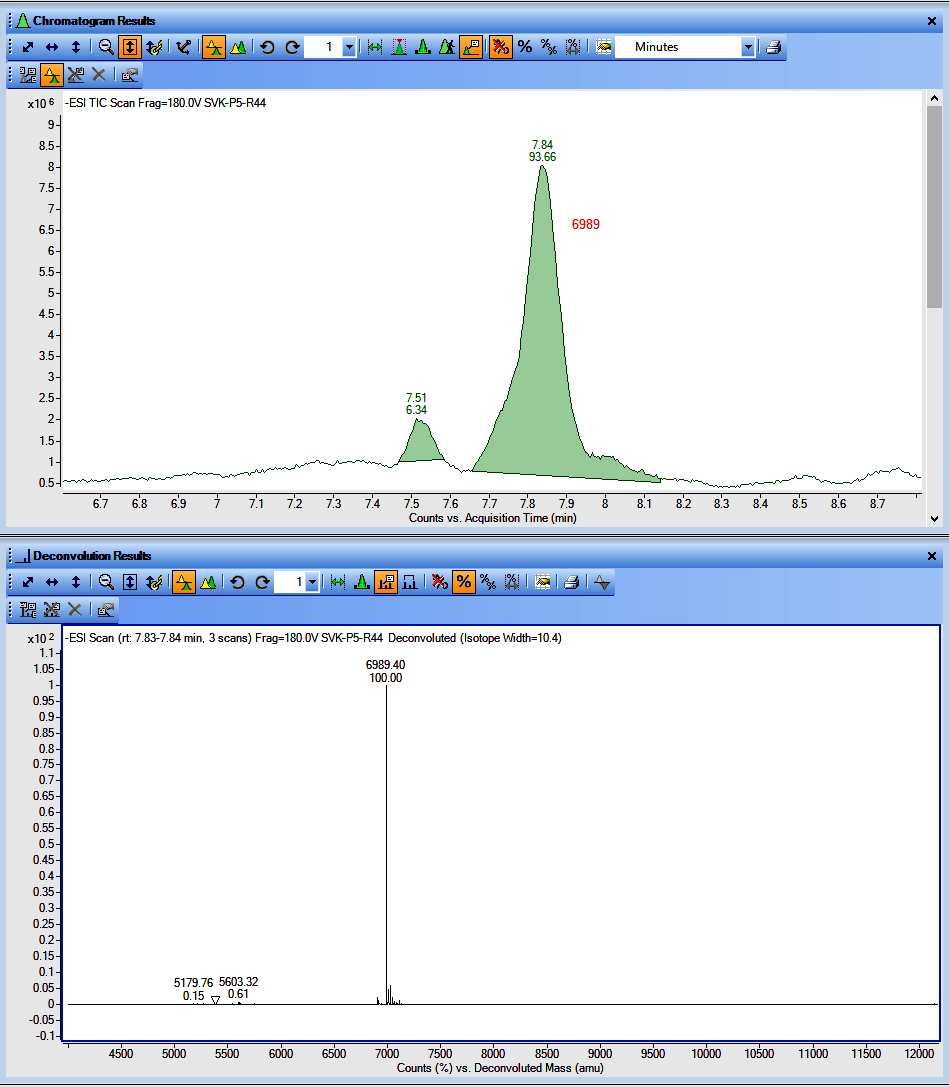
**

**Figure S37.** TIC and Deconvoluted mass spectrum of **3ai**, expected: 6989; observed 6989.

**
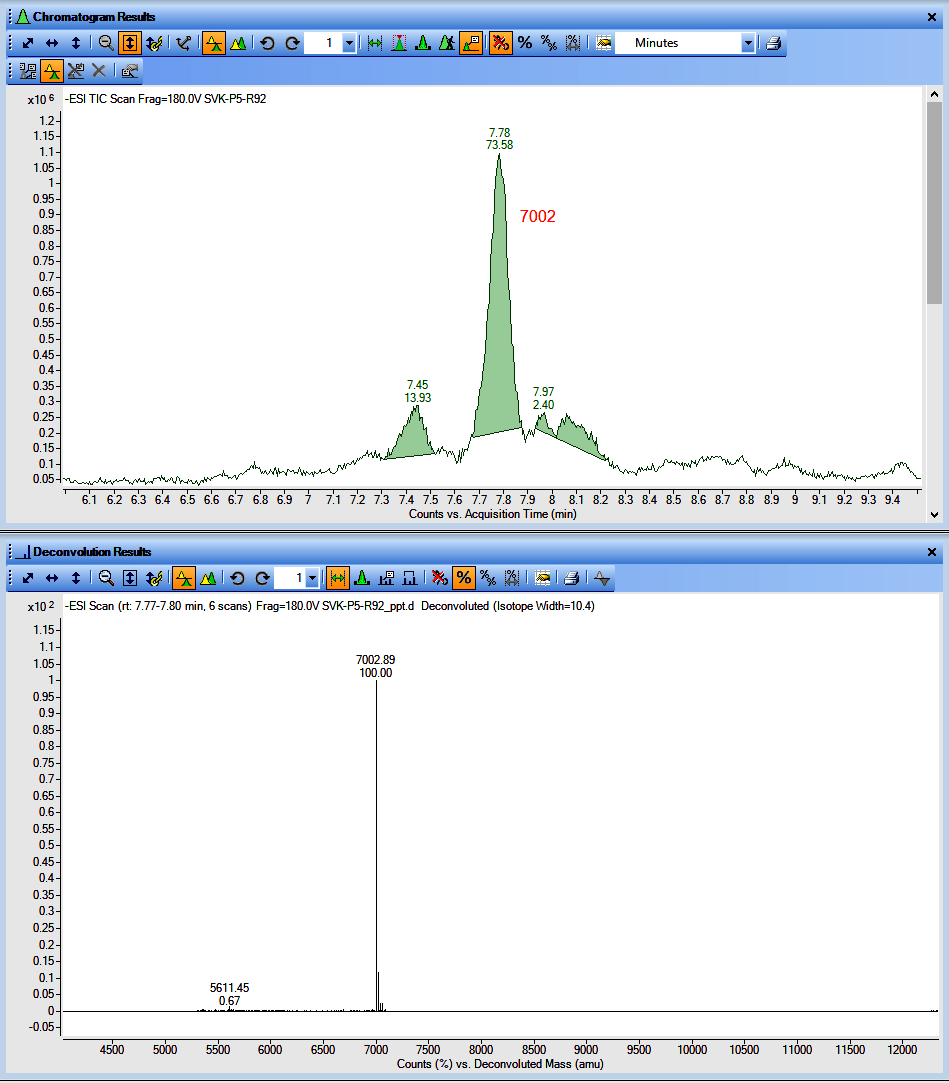
**

**Figure S38.** TIC and Deconvoluted mass spectrum of **3aj**, expected: 7002; observed 7002.

**
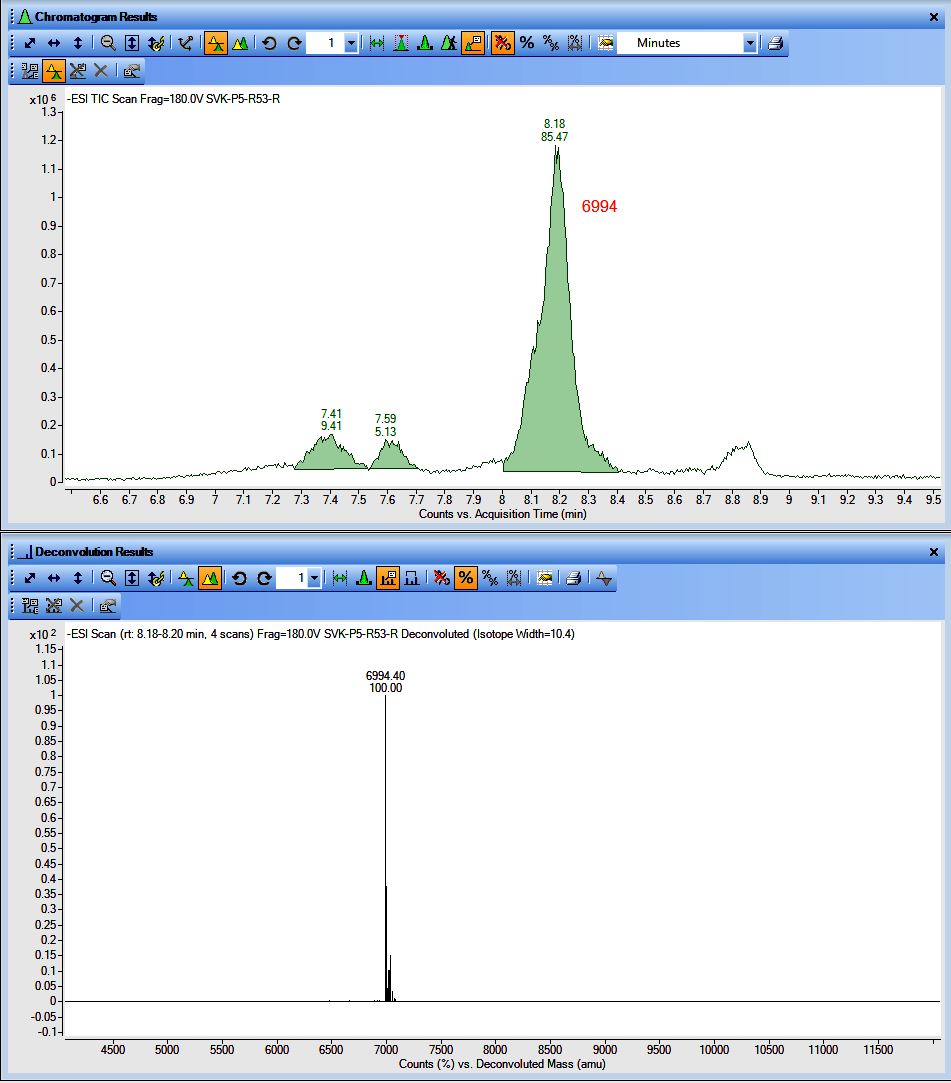
**

**Figure S39.** TIC and Deconvoluted mass spectrum of **3ak**, expected: 6994; observed 6994.

**
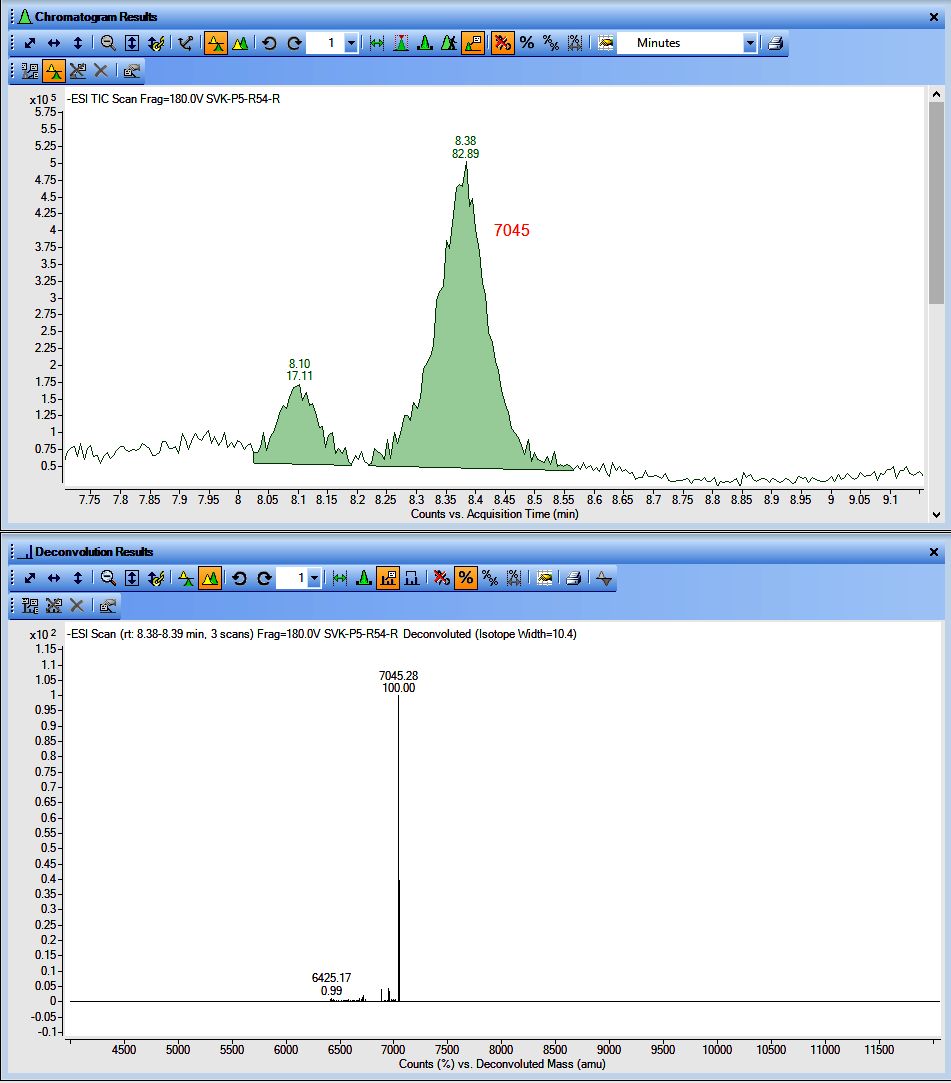
**

**Figure S40.** TIC and Deconvoluted mass spectrum of **3al**, expected: 7045; observed 7045.

**
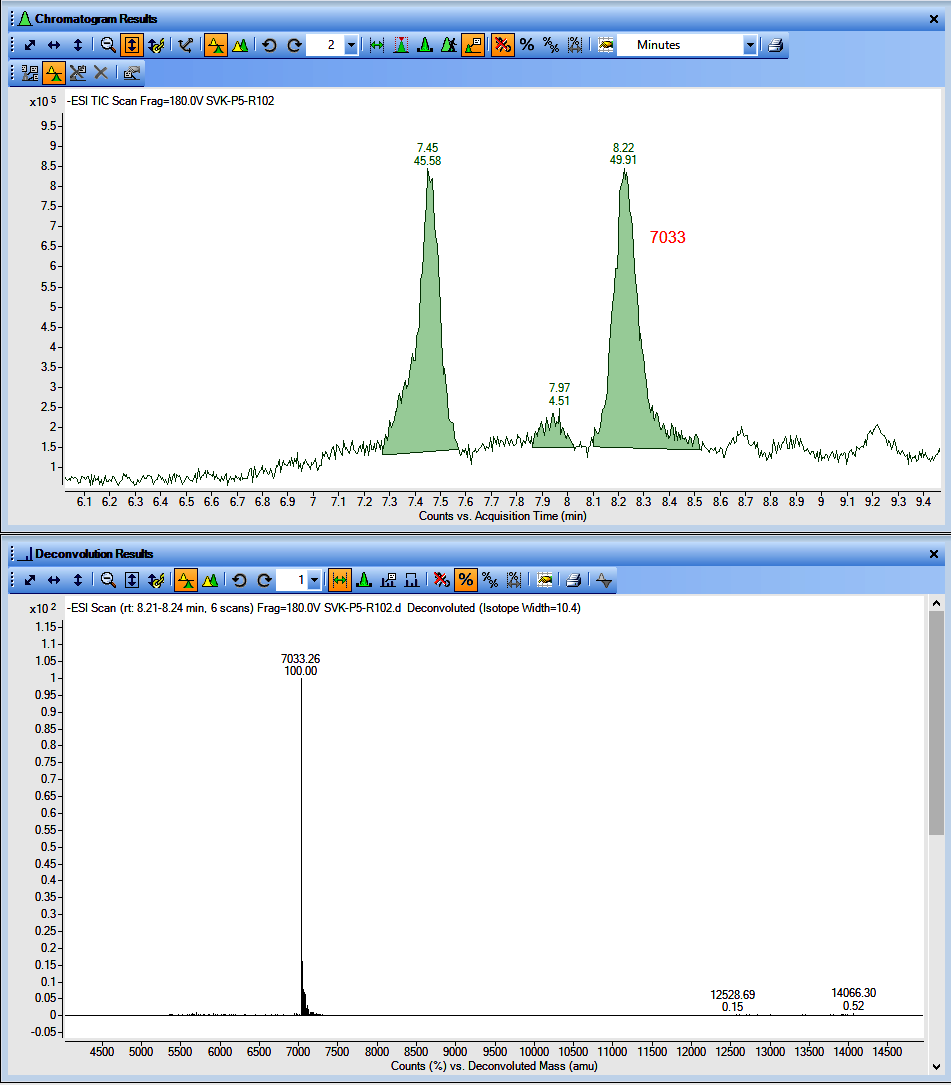
**

**Figure S41.** TIC and Deconvoluted mass spectrum of **3am**, expected: 7033; observed 7033.

**
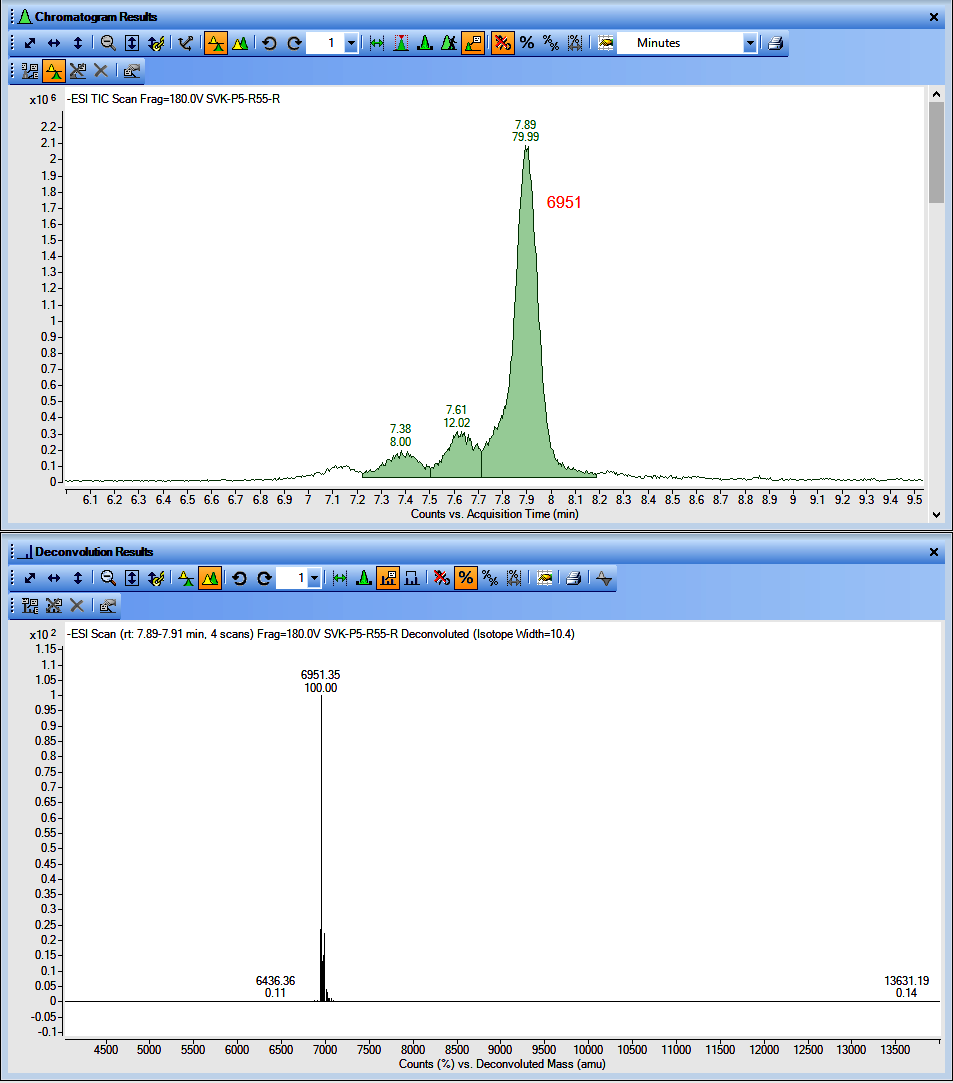
**

**Figure S42.** TIC and Deconvoluted mass spectrum of **5a**, expected: 6951; observed 6951.

**
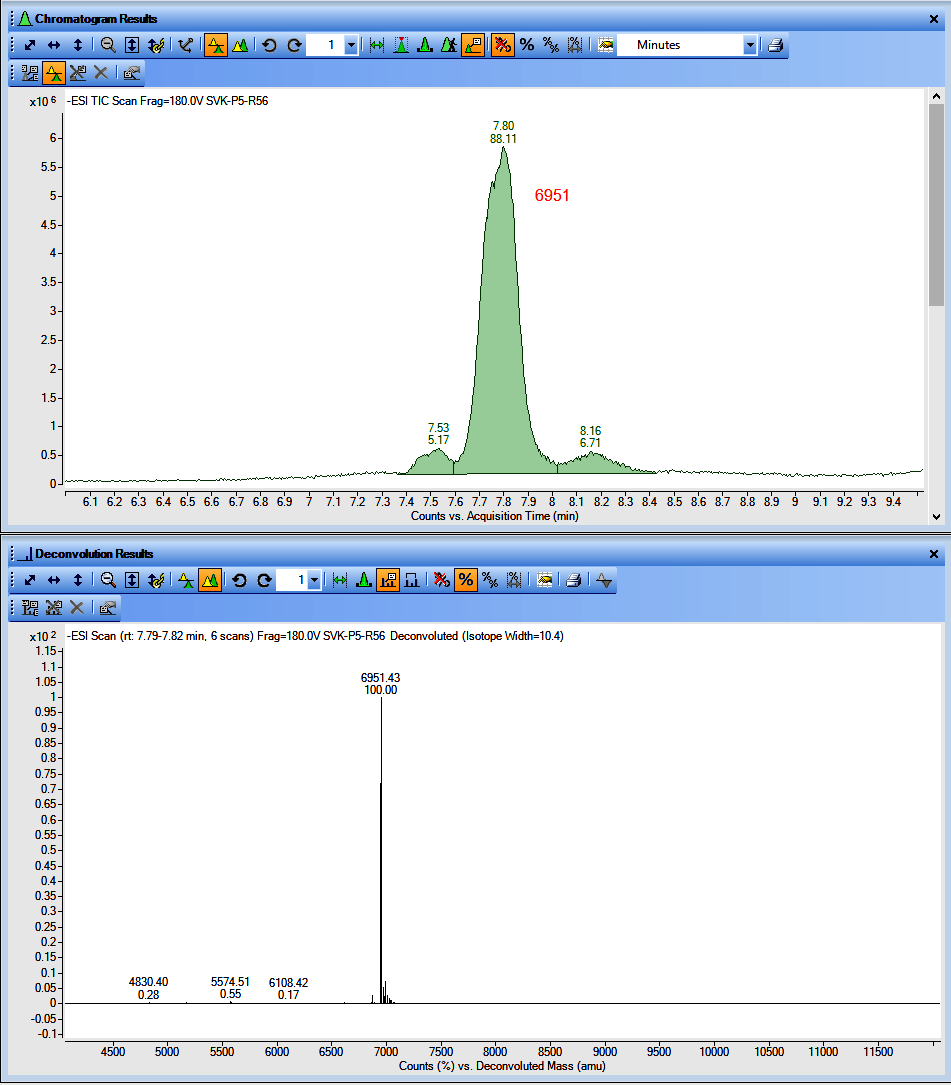
**

**Figure S43.** TIC and Deconvoluted mass spectrum of **5b**, expected: 6951; observed 6951.

**
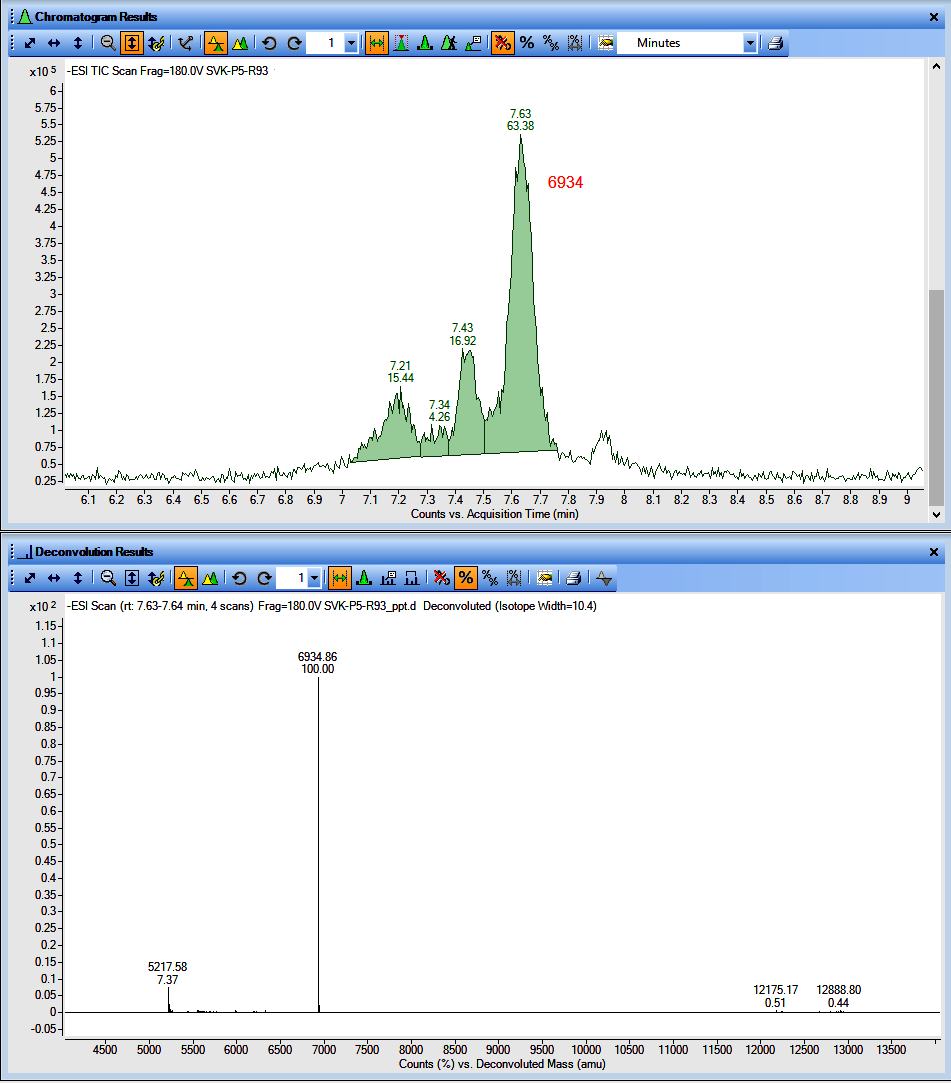
**

**Figure S44.** TIC and Deconvoluted mass spectrum of **5c**, expected: 6934; observed 6934.

**
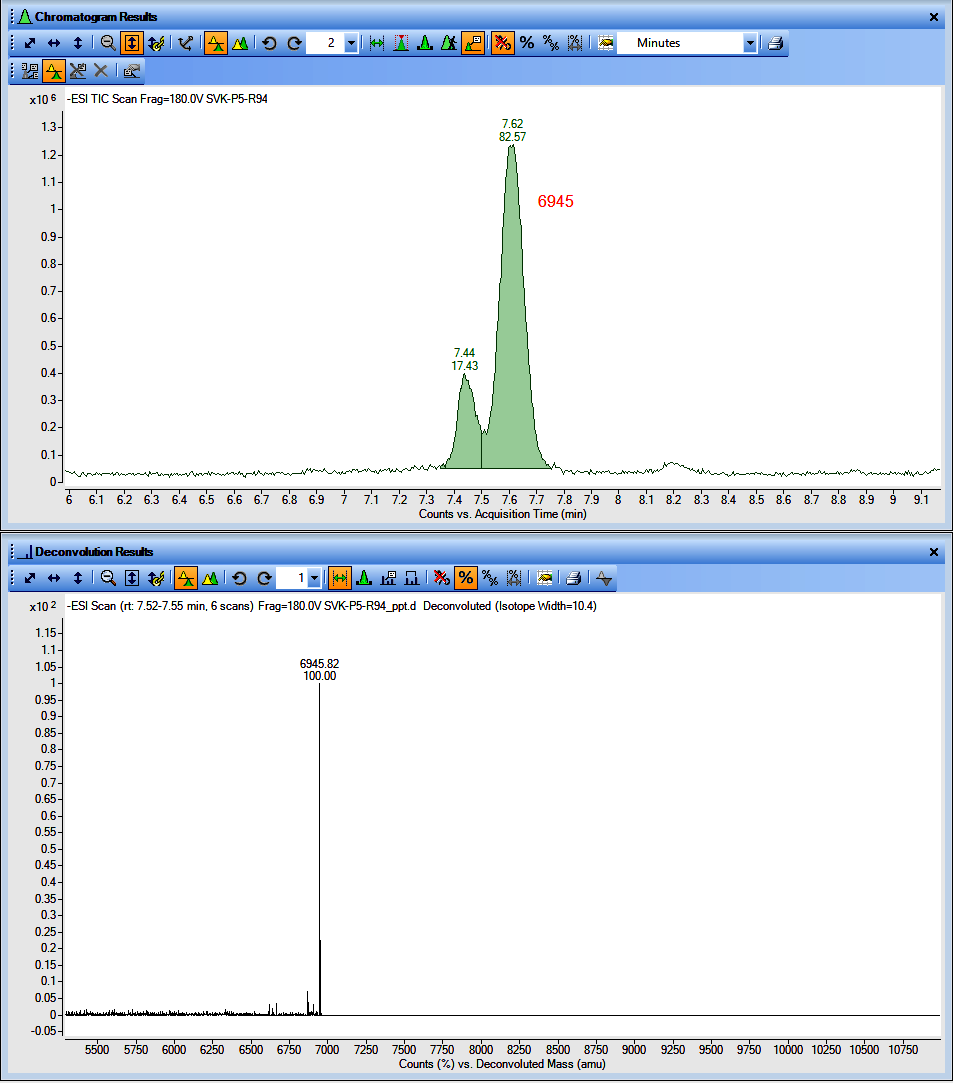
**

**Figure S45.** TIC and Deconvoluted mass spectrum of **5d**, expected: 6945; observed 6945.

**
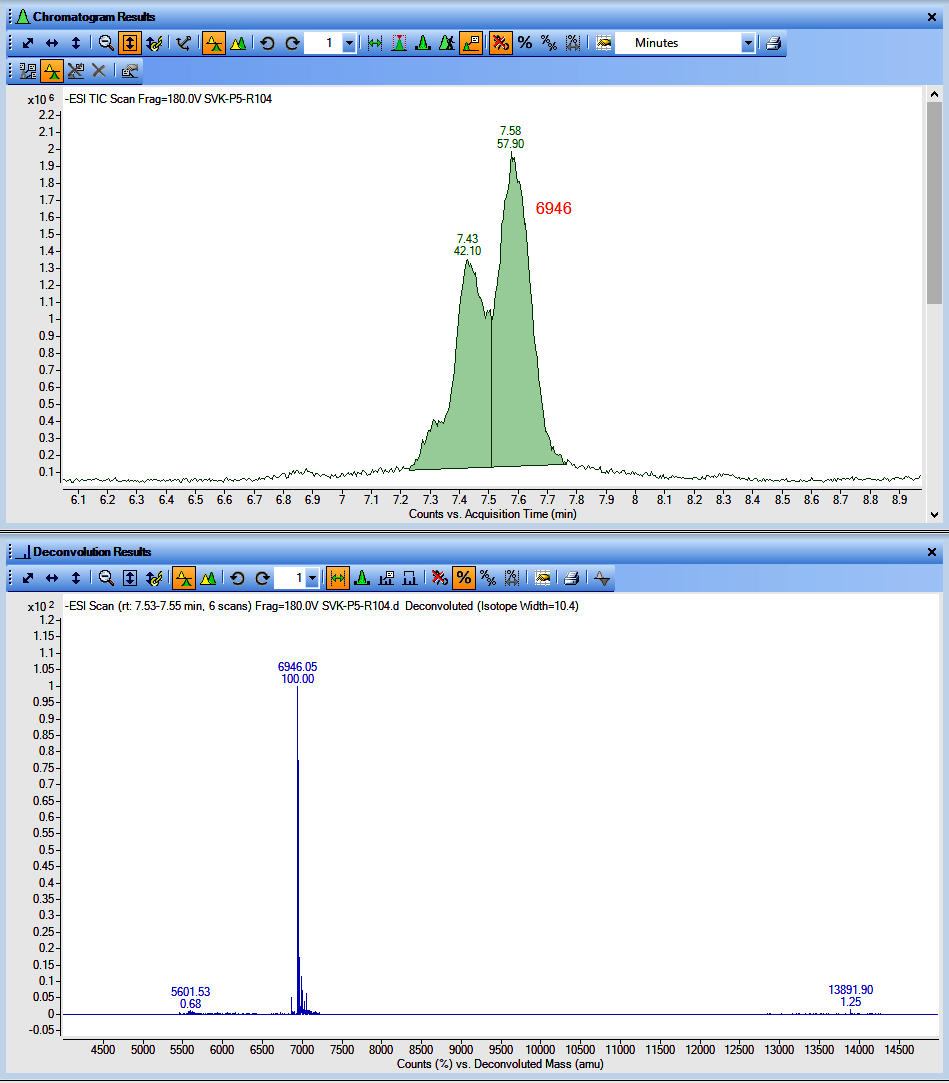
**

**Figure S46.** TIC and Deconvoluted mass spectrum of **5e**, expected: 6946; observed 6946.

**
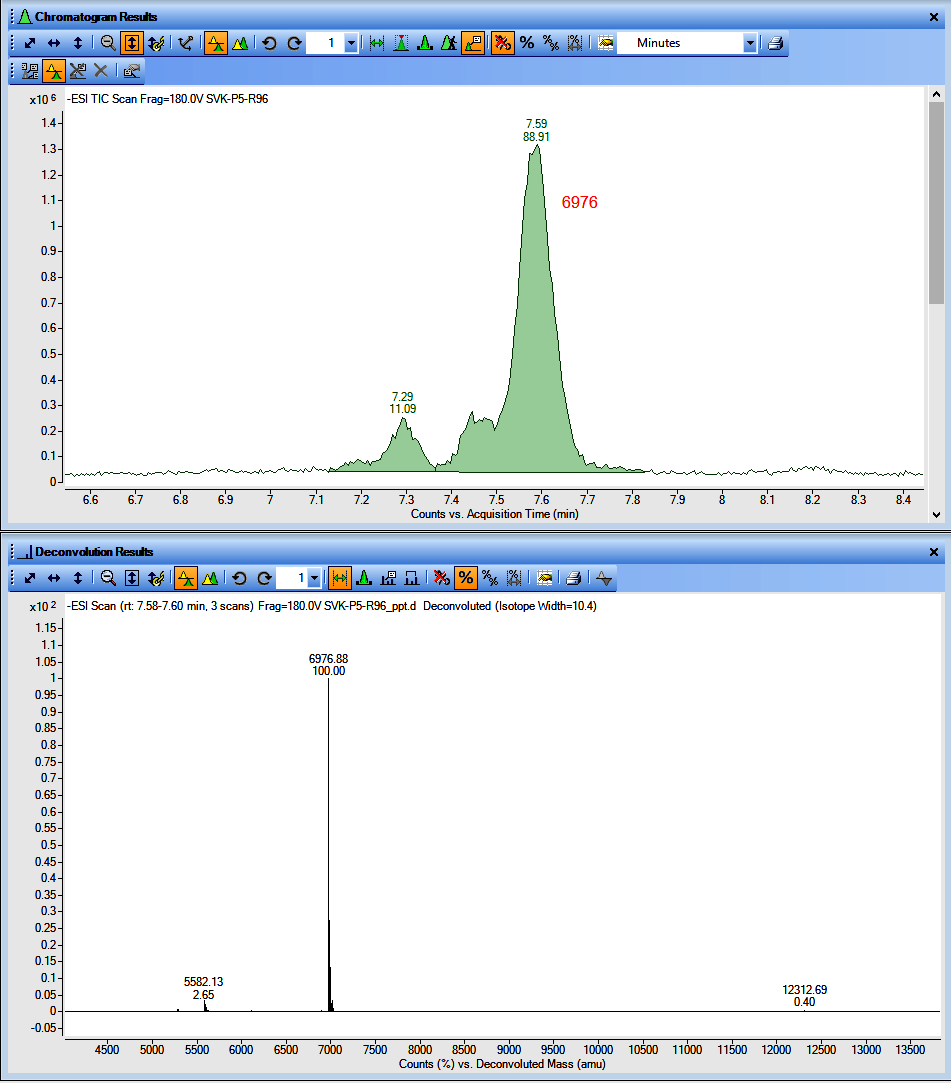
**

**Figure S47.** TIC and Deconvoluted mass spectrum of **5f**, expected: 6976; observed 6976.

**
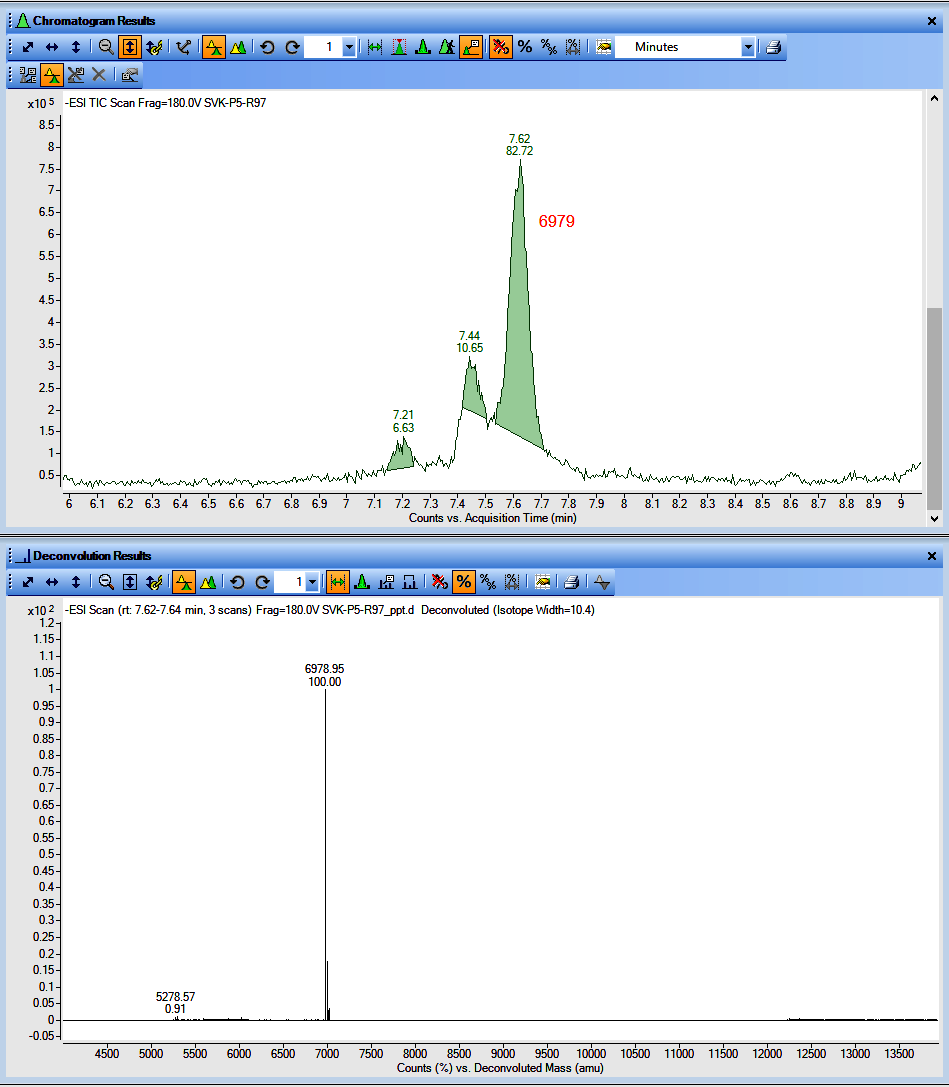
**

**Figure S48.** TIC and Deconvoluted mass spectrum of **5g**, expected: 6979; observed 6979.

**
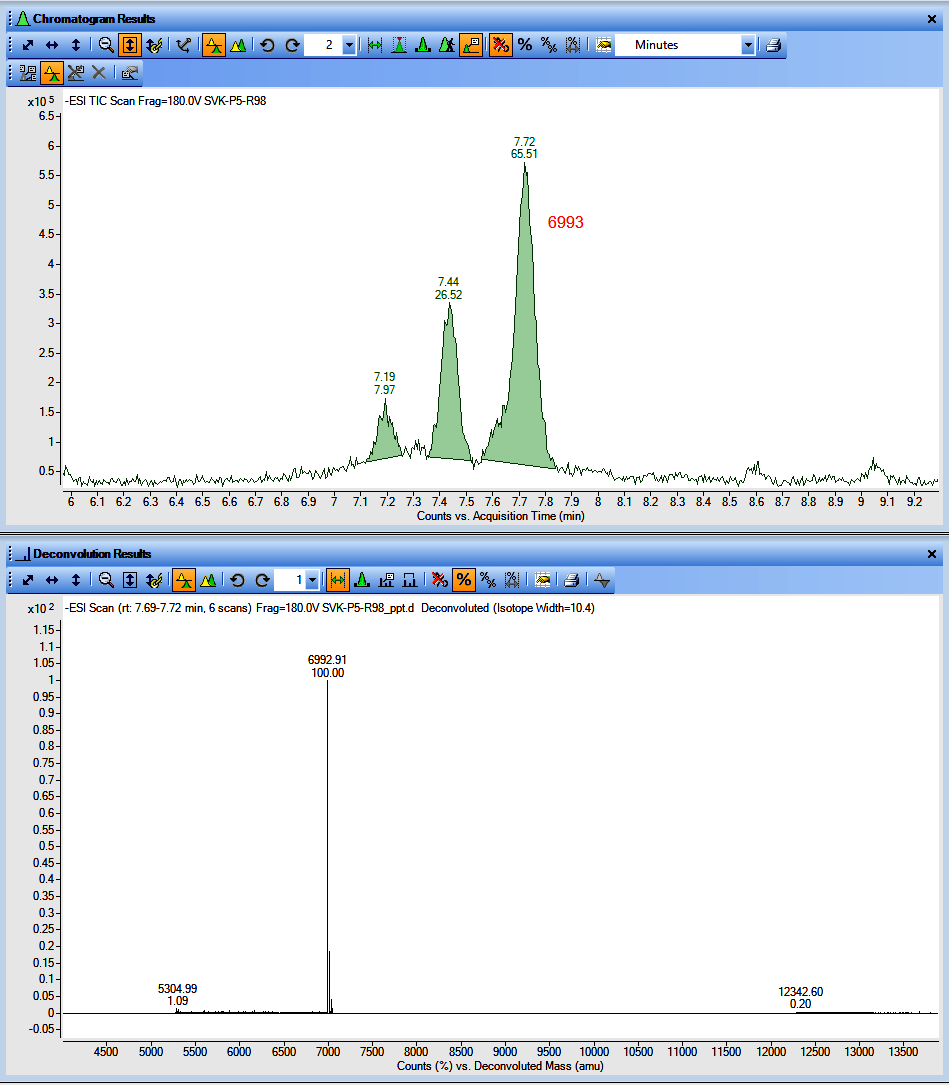
**

**Figure S49.** TIC and Deconvoluted mass spectrum of **5h**, expected: 6993; observed 6993.

**
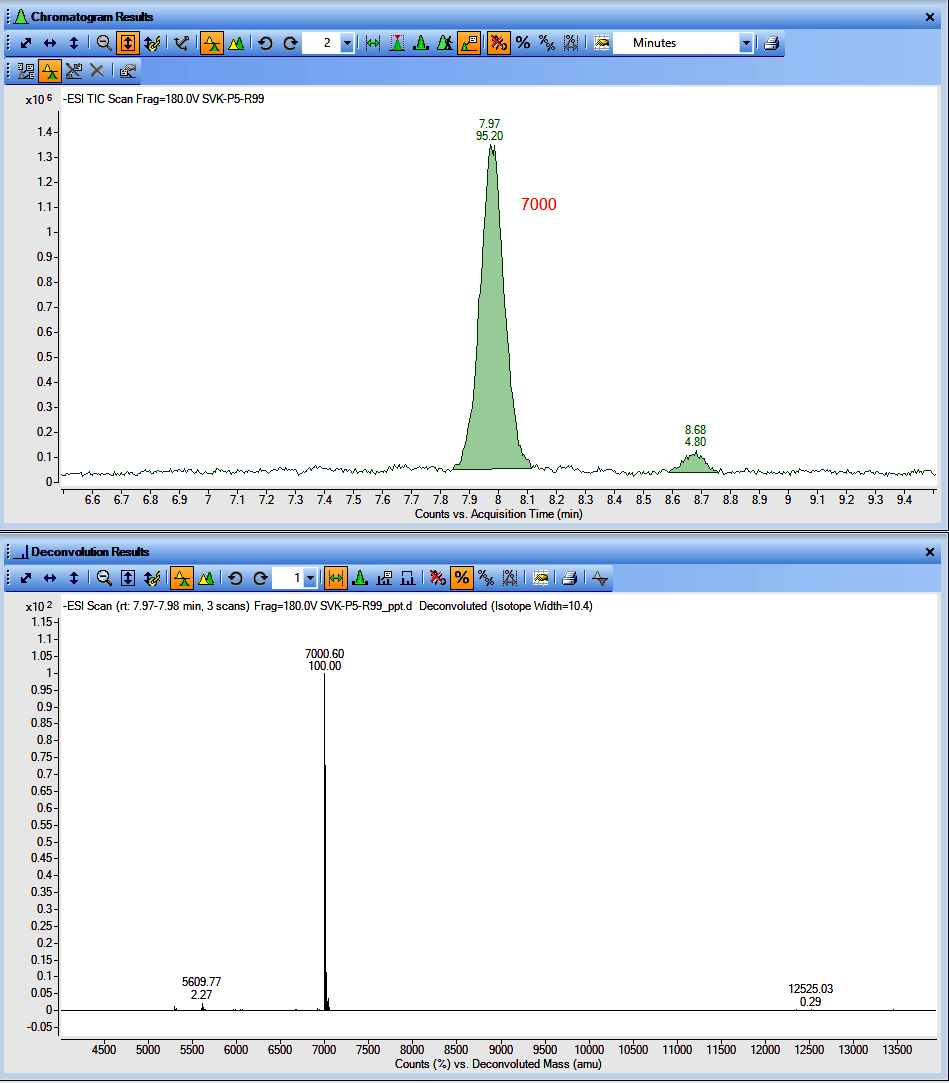
**

**Figure S50.** TIC and Deconvoluted mass spectrum of **5i**, expected: 7000; observed 7000.

**
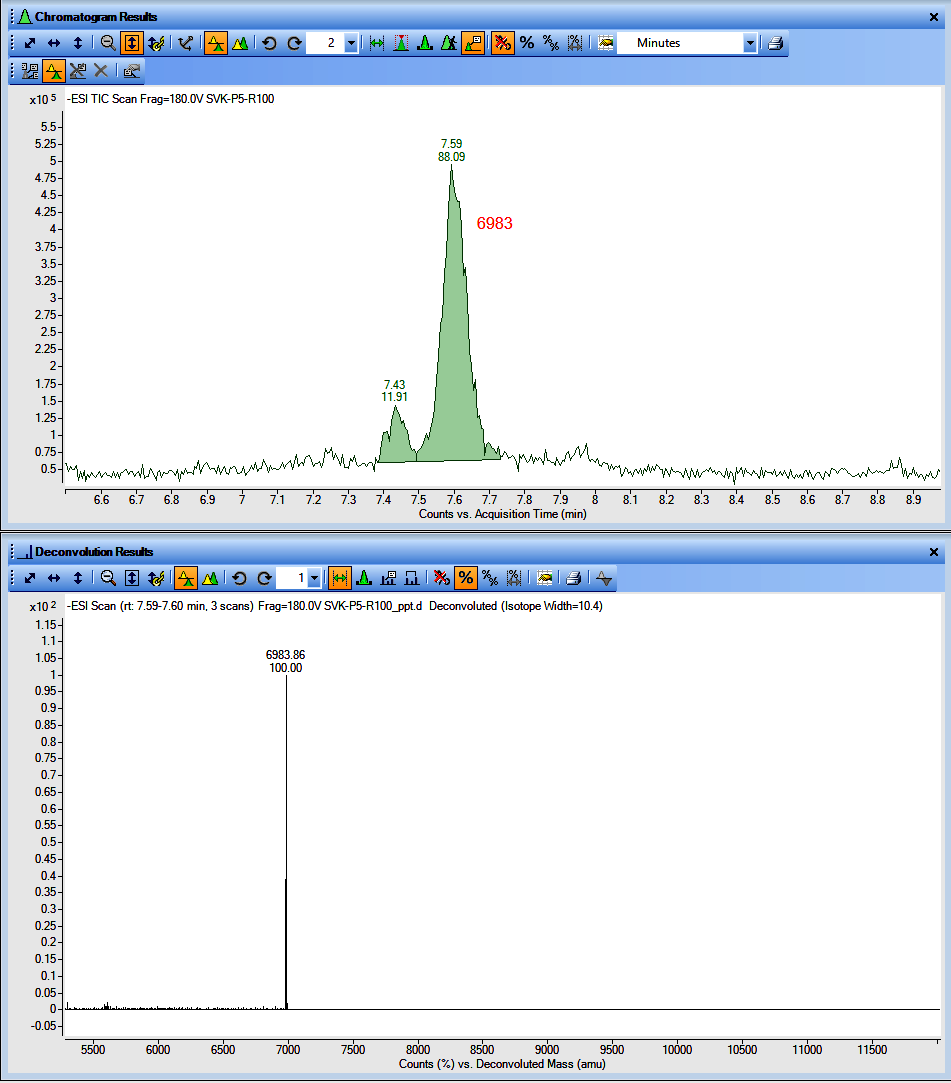
**

**Figure S51.** TIC and Deconvoluted mass spectrum of **5j**, expected: 6983; observed 6983.

**
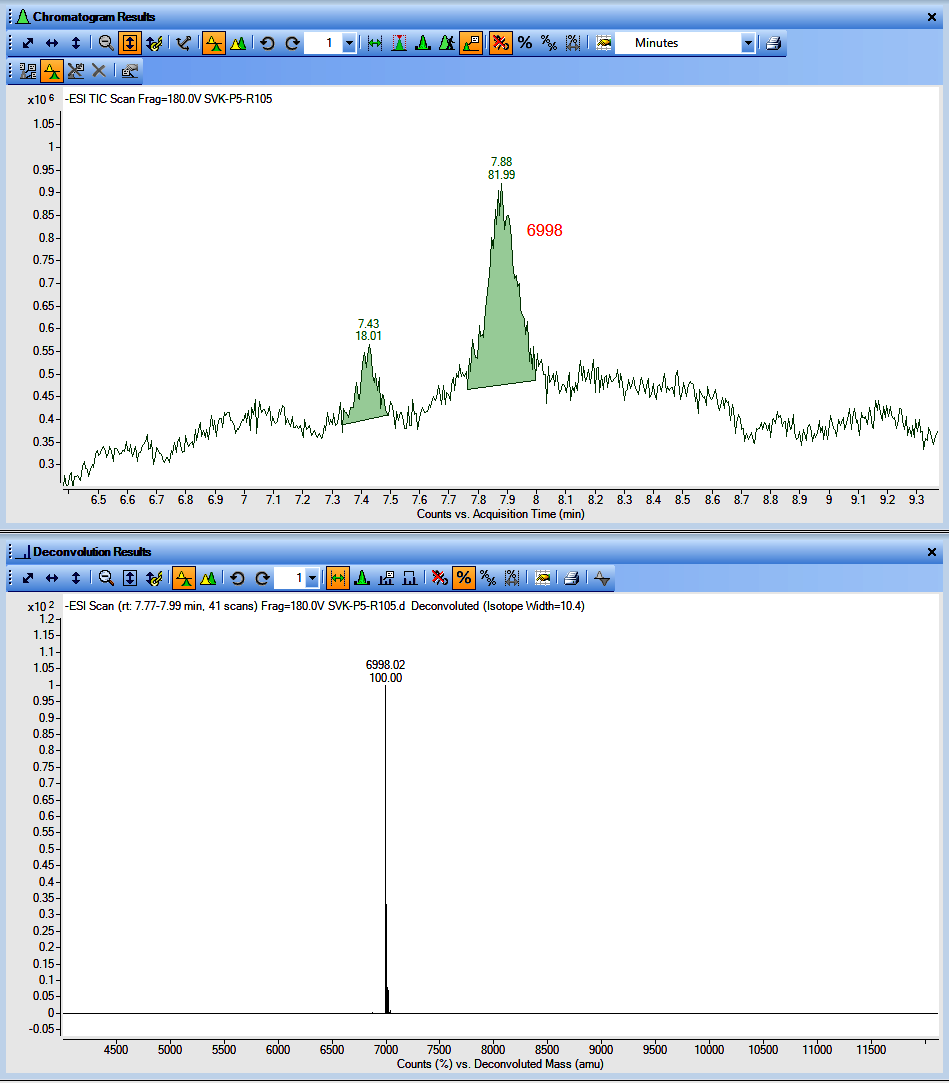
**

**Figure S52.** TIC and Deconvoluted mass spectrum of **5k**, expected: 6998; observed 6998.

**
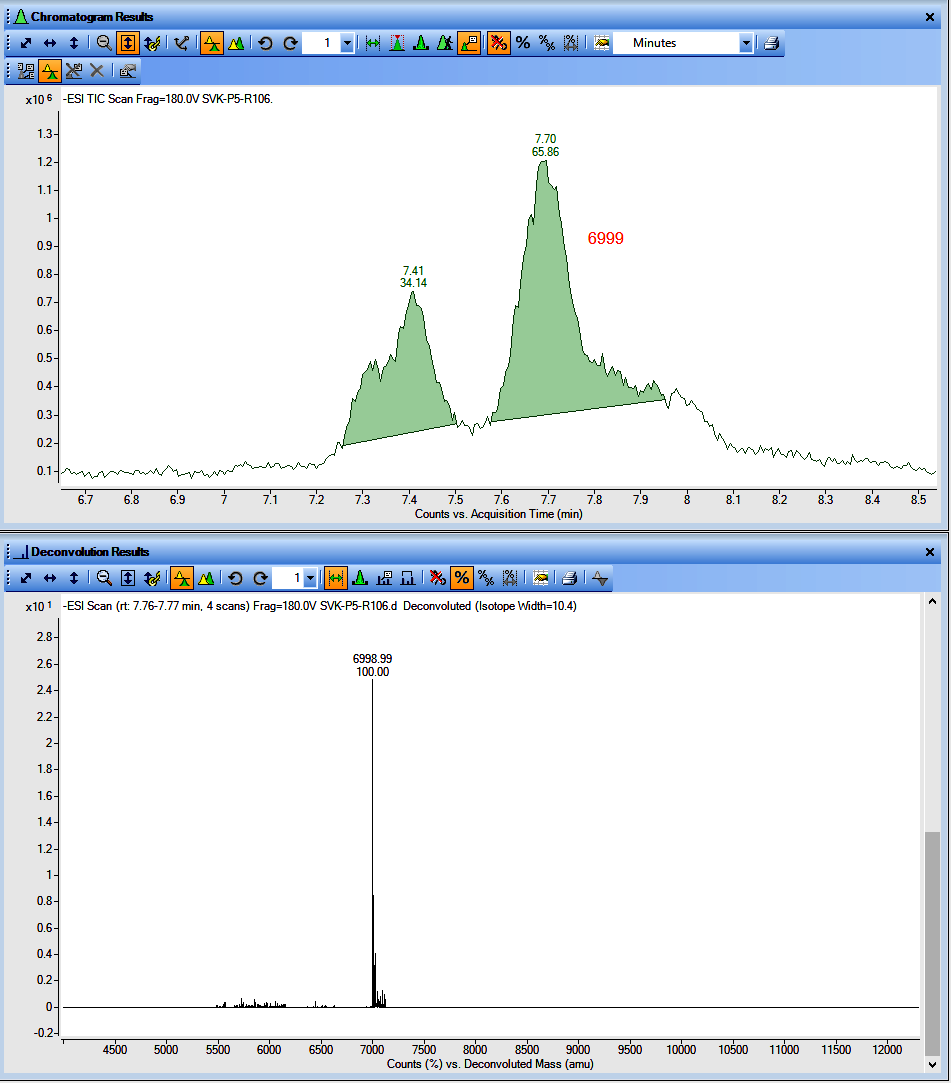
**

**Figure S53.** TIC and Deconvoluted mass spectrum of **5l**, expected: 6999; observed 6999.

**
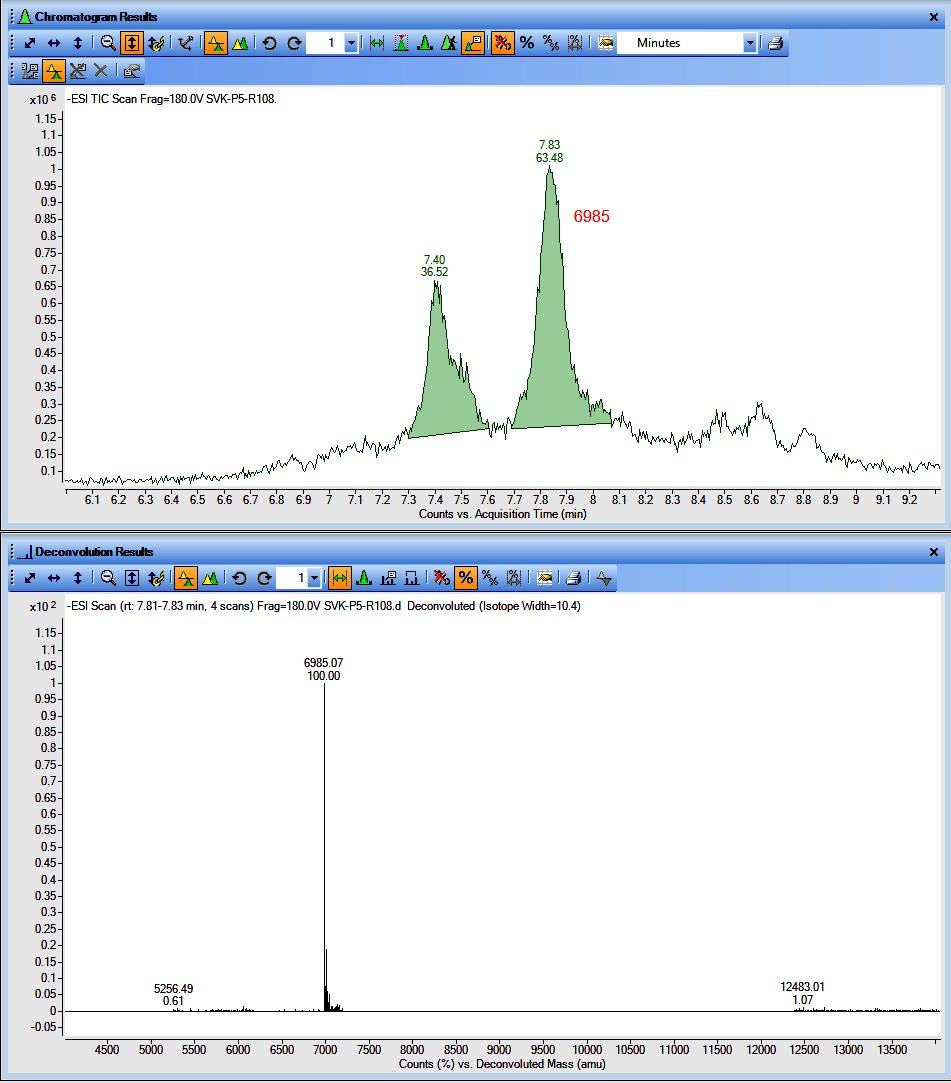
**

**Figure S524.** TIC and Deconvoluted mass spectrum of **5m**, expected: 6985; observed 6985.

**
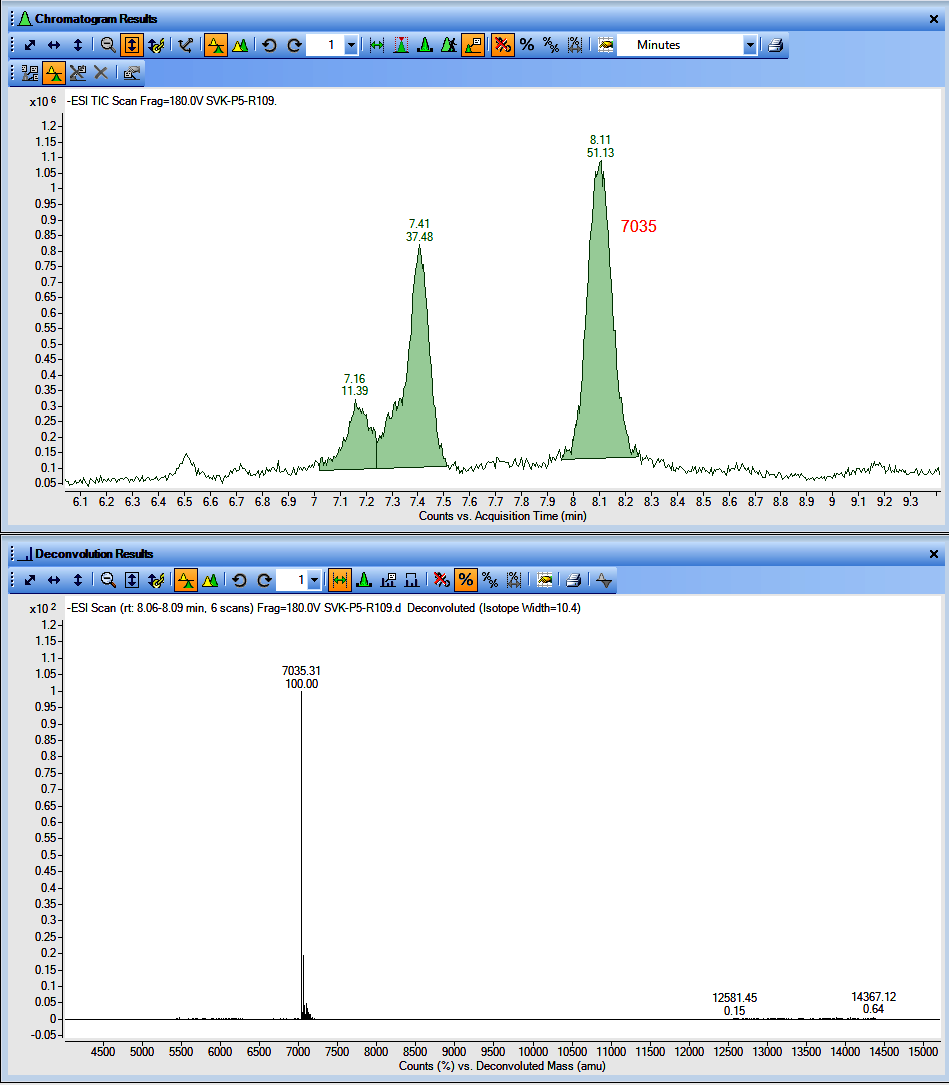
**

**Figure S55.** TIC and Deconvoluted mass spectrum of **5n**, expected: 7035; observed 7035.
